# Structure and dynamics of the proton-selective histidine and the gating tryptophan in an inward rectifying hybrid influenza B and A virus M2 proton channel†

**DOI:** 10.1039/d4cp01648c

**Published:** 2024-07-16

**Authors:** Yanina Pankratova, Matthew J. McKay, Chunlong Ma, Haozhou Tan, Jun Wang, Mei Hong

**Affiliations:** a Department of Chemistry, Massachusetts Institute of Technology 170 Albany Street Cambridge MA 02139 USA meihong@mit.edu; b Department of Pharmacology and Toxicology, College of Pharmacy, University of Arizona Tucson Arizona 85721 USA; c Department of Medicinal Chemistry, Rutgers University 160 Frelinghuysen Road Piscataway NJ 08854 USA

## Abstract

The M2 proteins of influenza A and B viruses form acid-activated proton channels that are essential for the virus lifecycle. Proton selectivity is achieved by a transmembrane (TM) histidine whereas gating is achieved by a tryptophan residue. Although this functional apparatus is conserved between AM2 and BM2 channels, AM2 conducts protons exclusively inward whereas BM2 conducts protons in either direction depending on the pH gradient. Previous studies showed that in AM2, mutations of D44 abolished inward rectification of AM2, suggesting that the tryptophan gate is destabilized. To elucidate how charged residues C-terminal to the tryptophan regulates channel gating, here we investigate the structure and dynamics of H19 and W23 in a BM2 mutant, GDR-BM2, in which three BM2 residues are mutated to the corresponding AM2 residues, S16G, G26D and H27R. Whole-cell electrophysiological data show that GDR-BM2 conducts protons with inward rectification, identical to wild-type (WT) AM2 but different from WT-BM2. Solid-state NMR ^15^N and ^13^C spectra of H19 indicate that the mutant BM2 channel contains higher populations of cationic histidine and neutral τ tautomers compared to WT-BM2 at acidic pH. Moreover, ^19^F NMR spectra of 5-^19^F-labeled W23 resolve three peaks at acidic pH, suggesting three tryptophan sidechain conformations. Comparison of these spectra with the tryptophan spectra of other M2 peptides suggests that these indole sidechain conformations arise from interactions with the C-terminal charged residues and with the N-terminal cationic histidine. Taken together, these solid-state NMR data show that inward rectification in M2 proton channels is accomplished by tryptophan interactions with charged residues on both its C-terminal and N-terminal sides. Gating of these M2 proton channels is thus accomplished by a multi-residue complex with finely tuned electrostatic and aromatic interactions.

## Abstract 2

Solid-state NMR spectra of a mutant influenza B M2 protein shine light on how charged residues near a tryptophan impact proton shuttling by a key histidine, thus regulating the proton conduction direction in this family of proton channels.

## Graphical abstract

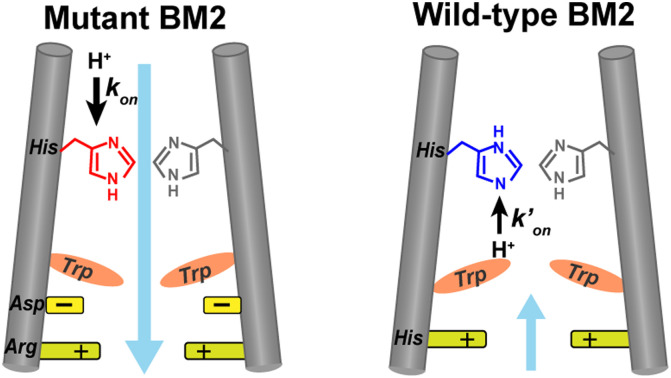

## 1. Introduction

The M2 proteins of the influenza A and B viruses (AM2 and BM2) are small viroporins that conduct protons across the lipid membrane.^1–3^ This proton channel function is activated by acidic pH^4^ and is important for the virus lifecycle.^5^ Proton conduction by M2 is responsible for acidifying the endosomally entrapped virus to initiate viral uncoating. In certain subtypes of the influenza A virus, the M2 proton channel activity also equilibrates the pH of the *trans*-Golgi apparatus and the cytoplasm to prevent premature conformational changes of hemagglutinin.^5^ Both AM2 and BM2 contain a single transmembrane (TM) helix that tetramerizes to form a proton-conducting pore in lipid bilayers.^6,7^ The TM peptide of AM2 (residues 22–46) shows channel activity that is within a factor of two of the full-length protein,^8^ whereas the TM peptide of BM2 (residues 1–33) exhibits the same activity as the full-length protein.^9^ Thus, the TM domain constitutes the functional core of M2 proton channels.

**Fig. 1:**
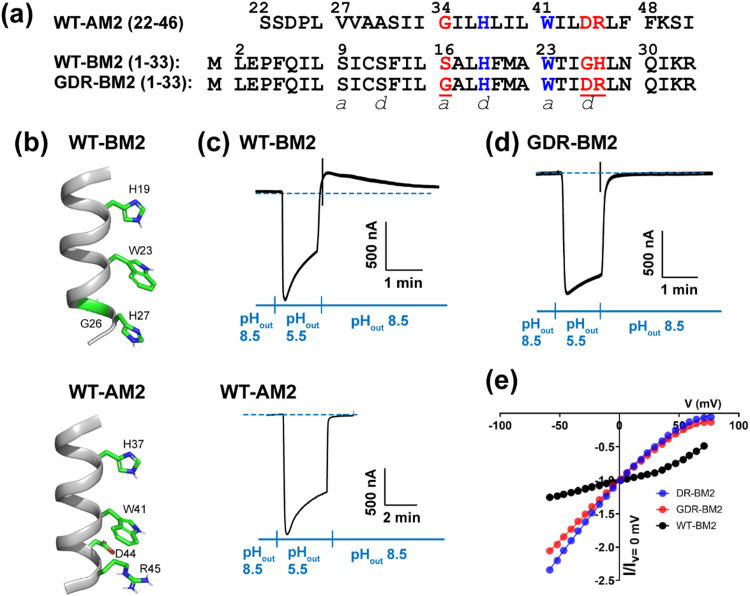
Amino acid sequence and proton channel activity of GDR-BM2. (a) Amino acid sequences of the TM domains of WT-AM2, WT-BM2 and GDR-BM2 proteins. The sequences are shown as heptad repeats, where the positions of the pore-facing *a* and *d* are indicated. The functional H and W residues are colored in blue while the mutated residues in GDR-BM2 and their corresponding residues in the WT proteins are shown in red. (b) Cartoon of the positions of the functional residues in WT-BM2 and WT-AM2. WT-BM2 contains a symmetric HxxxWxxxH motif whereas WT-AM2 contains a HxxxW motif followed by D44 and R45. (c) Representative recording traces of full-length WT-BM2 and WT-AM2 in oocytes. Both proteins conduct inward proton current under low pH_out_. BM2 additionally shows weak outward current under low pH_in_ while AM2 does not. (d) Representative recording traces of full-length GDR-BM2 in oocytes. The mutant shows the same inward rectification as WT-AM2. (e) Current–voltage (*I*–*V*) relationship of WT-BM2, DR-BM2, and GDR-BM2. A voltage ramp from −60 to 80 mV was applied after the oocyte displayed maximum inward current at pH 5.5. pH 8.5 background current was subtracted from pH 5.5 current. WT-BM2 displays outward rectified *I*–*V* curve while DR-BM2^9^ and GDR-BM2 display slightly inward rectified *I*–*V* curve.

Extensive biochemical and biophysical studies show that the proton conduction properties of AM2 and BM2 are similar in some respects while different in others.^3,10^ Both proteins conduct protons down the concentration gradient, but AM2 conducts protons exclusively from the N-terminus to the C-terminus (inward direction) when the external pH (pH_out_) is low but cannot conduct protons from the C-terminus to the N-terminus when the internal pH (pH_in_) is low. In contrast, BM2 is able to conduct protons in either direction depending on the pH gradient on the two sides of the membrane.^9^ The conductance of BM2 (1–33) is two-fold larger than that of AM2 (18–60).^11^ Both AM2 and BM2 use a pore-facing histidine to select for protons^4^ and a pore-facing tryptophan for channel gating.^12^ These two residues are separated by one helical turn and form a conserved HxxxW motif in all AM2 and BM2 variants (Fig. 1(a)). Apart from this HxxxW motif, AM2 and BM2 have low sequence homology. The AM2 pore is lined by predominantly nonpolar residues (V27, A30, and G34) whereas the BM2 pore is lined by polar residues such as S9, S12 and S16. The residues that interact with tryptophan from the C-terminal side also differ: in AM2, W41 is flanked by a negatively charged D44 and a positively charged R45 on its C-terminal side, whereas in BM2, W23 is in contact with G26 and H27 at the same positions (Fig. 1(b)).

Interactions between the aromatic and charged residues in the C-terminal region of the M2 TM domain have been studied before. Cation–π interactions between histidine and tryptophan were first reported by Raman spectroscopy for AM2^13^ and BM2^14^ and subsequently investigated for AM2 in atomic detail using solid-state NMR.^15,16^ Crystal structures of AM2 show that the His–Trp interaction is stabilized by water-mediated hydrogen bonds between the D44 sidechain and the W41 indole nitrogen,^17^ and may be further stabilized by an interhelical salt bridge between D44 and R45.^18^ D44N, D44A, and D44C mutations abolished inward rectification in AM2, suggesting a loosening of the channel gate.^17^ Consistent with these functional data, solution NMR spectra and molecular dynamics simulations indicate that Trp and its surrounding residues become more disordered in these D44 mutants compared to wild-type AM2. Since WT-BM2 contains a G26 at the equivalent position of D44, a G26D, H27R-BM2 mutant (DR-BM2) was engineered to assess the impact of these C-terminal TM residues on BM2's proton conduction.^9^ Channel current data showed that DR-BM2 fully inhibited the reverse current under acidic pH_in_, similar to WT AM2. In comparison, a single G26D mutation reduced the reverse current by 50% while a single H27R mutation did not attenuate the reverse current. These data thus indicate that the aspartate C-terminal to the tryptophan is more potent than the arginine in tightening the channel gate. Cation–π interactions have also been reported between R45 and F48 based on the crystal structure of V27A-AM2 (21–61),^19^ although the significance of this interaction for channel gating is not known.

Atomic-level insights into the proton conduction mechanisms of influenza M2 proteins have been obtained from solid-state NMR (ssNMR) studies of the histidine structure and dynamics.^10,20–23^^15^N and ^13^C chemical shifts of the histidine imidazole ring are exquisitely sensitive to its charge and tautomeric state. By measuring the ^15^N and ^13^C chemical shifts as a function of pH, we established that the proton-selective H19 in BM2 protonates at lower pH^24^ compared to the proton-selective H37 in AM2.^20,22^ The lower p*K*_a_'s of BM2 H19 were subsequently found to be linked to the presence of H27 at the C-terminal end of the TM domain: an H27A mutation shifted the H19 equilibria towards the cationic state, approaching the H37 equilibria in AM2.^25^ NMR chemical shifts also revealed that a W41F mutation in AM2 permitted protonation of H37 from the C-terminal side^26^ when the N-terminal pore is blocked by amantadine.^27^ These data provided direct structural evidence for reverse (C-terminal) protonation of histidine in a gate-deficient mutant channel, supporting channel activity data.^12^

Insights into the global structural features that support proton conduction by M2 proteins came from high-resolution structure determination. X-ray crystal structures of AM2 in detergents and lipid cubic phases^18,28,29^ revealed that AM2 adopts two main conformations: at neutral pH the protein exhibits a C_closed_ conformation that has a relatively tight C-terminal pore, whereas at acidic pH the protein adopts a C_open_ conformation that is characterized by a wide C-terminal pore. These two distinct conformations suggest that AM2 behaves like a transporter, alternately opening to the two sides of the membrane. This model is consistent with inward rectification of AM2: even if the pH of the C-terminal interior (pH_in_) is low, as long as the pH of the N-terminal exterior (pH_out_) is high, the C_closed_ conformation would dominate, thus prohibiting outward proton conduction. The transition between the two conformations could be partly facilitated by a glycine, G34, in the middle of the AM2 TM domain. Oriented-membrane ssNMR data and chemical shift analyses showed that this G34 introduces a kink to the TM helix under certain conditions.^30,31^

In contrast to AM2, high-resolution ssNMR structures of membrane-bound BM2 do not exhibit transporter-like conformational changes.^32^ At both neutral and acidic pH, the BM2 TM helix is unkinked, but the helix is ∼6° more tilted from the bilayer normal at acidic pH than at neutral pH. The unkinked conformation of the helix is consistent with the fact that the central residue in the BM2 TM domain is not a glycine but a serine (S16), which cannot cause a kink. The BM2 chemical shifts also do not change with pH, indicating that the backbone conformation is unchanged, in contrast to the pH dependence of AM2 chemical shifts. These results led to the proposal that the BM2 proton channel activates at acidic pH using a scissor-like mechanism. This symmetric activation mechanism is qualitatively consistent with the bidirectional proton conduction of the channel.

These biophysical and biochemical data suggest that a BM2 mutant that has two charged residues C-terminal to the tryptophan and a conformationally plastic Gly in the middle of the TM domain might tighten the channel gate and increase the propensity of BM2 helix to kink. In this study, we report channel activity data and solid-state NMR data of such a mutant, GDR-BM2, in which three native BM2 residues are replaced by the corresponding AM2 residues: S16G, G26D, and H27R. We measured ^15^N and ^13^C NMR spectra of the proton-selective H19 and ^19^F NMR spectra of the gating W23 in membrane-bound GDR-BM2. These spectra were measured at neutral and acidic pH and were compared with previously reported NMR spectra of various M2 peptides. These results provide new insight into how charged residues in the C-terminal TM region of M2 proteins allosterically regulate the proton transfer equilibria of the proton-selective histidine *via* the gating tryptophan.

## 2. Experimental section

### 2.1 Solid-phase peptide synthesis of site-specifically labeled GDR-BM2 (1–33)

GDR-BM2 (1–33) was synthesized using Fmoc solid-phase peptide synthesis (SPPS) on a home-built fast-flow synthesizer.^33^ The amino acid sequence is MLEPFQILSI SSFILG̲ALHF MAWTID̲R̲LNQ IKR, which includes S16G, G26D, H27R mutations on the influenza B/Maryland/1/2001 M2 (1–33) sequence. S9, H19, and I25 were labeled with ^13^C and ^15^N and W23 was 5-^19^F-labeled. H-Rink amide ChemMatrix® resin at 0.050 mmol (0.10 g at 0.5 mmol g^−1^ loading size) was loaded into the reactor, which was maintained at 70 °C. Unlabeled amino acids were coupled in ten-fold excess (0.50 mmol) for 45 s whereas labeled amino acids were coupled in four-fold excess (0.20 mmol) for 68 s. To reduce single-residue deletion impurities at labeled residues, a second coupling using unlabeled amino acid was performed immediately after the coupling of the labeled amino acid. H19 and H27 were manually coupled at room temperature for 30 min to reduce racemization during coupling at high temperatures.^34^ After the final coupling step, the resin was dried, and the peptide was deprotected and cleaved from the resin with 5 mL of trifluoroacetic acid (TFA)/phenol/water/triisopropylsilane (TIPS) solution (88 : 5 : 5 : 2 by volume) for 2 h. The resin was filtered off, and the crude peptide was precipitated from the cleavage solution with cold diethyl ether, washed twice, then dried under vacuum overnight. The crude peptide was dissolved in a 50% acetonitrile (ACN)–water mixture and purified by preparative reverse-phase high-performance liquid chromatography (RP-HPLC) on a Varian Prostar 210 System using an Agilent Zorbax C3 column (5-μm particle size, 21.2 mm  ×  150 mm). Purification was conducted using a linear gradient of 40–55% ACN over 25 min at a flow rate of 10 mL min^−1^. Fractions containing the peptide were lyophilized. Matrix-assisted laser desorption ionization mass spectrometry (MALDI-MS) analysis confirmed the mass of the pure product to be 3875.1 Da, which matches the calculated mass of 3875.06 Da. Final purity was confirmed by analytical RP-HPLC.

### 2.2 Electrophysiological experiments of GDR-BM2

Full-length AM2 and BM2 sequences from influenza A/Udorn/72 and influenza B/Lee/40 viruses were cloned into a pGEM3 vector. GDR-BM2 was generated by site-directed mutagenesis using site overlap extension polymerase chain reaction according to the QuikChange (Agilent) mutagenesis manual. pGEM3 plasmids encoding AM2, BM2, or GDR-BM2 were linearized by digesting the Hind III restriction site downstream of the gene, and *in vitro* transcription reactions were performed on the linearized DNA by using a T7 mMESSAGE mMACHINE transcription kit (Ambion). *Xenopus laevis* oocytes were prepared, injected, and maintained as described before.^35^ The *Xenopus* frog colony maintenance and oocyte harvesting comply with all relevant ethical regulations of the United States. The frogs were maintained at the University of Arizona university animal care facility and all animal experiments were approved by the University of Arizona Institutional Animal Care and Use Committee (IACUC) with approved protocol # 14-524. Whole cell current was recorded from oocytes 24–72 h after mRNA injection using a two-electrode voltage clamp technique as described before.^9^ For measuring the *I*–*V* relationship, a voltage ramp ranging from −60 to 80 mV was applied when oocytes displayed maximum inward current, and the duration of the voltage ramp was 2 s. Background current at pH 8.5 was subtracted when we plotted the *I*–*V* curves.

### 2.3 Membrane sample preparation

Purified GDR-BM2 (1–33) peptide was reconstituted into a virus-mimetic lipid membrane (VM+) containing equimolar amounts of 1-palmitoyl-2-oleoyl-*sn-glycero*-3-phosphocholine (POPC), 1-palmitoyl-2-oleoyl-*sn-glycero*-3-phospho-ethanolamine (POPE), sphingomyelin (SM) and cholesterol. The protein to total lipid molar ratio was 1 : 16. This membrane immobilizes the peptide backbone over a broad temperature range, thus allowing us to detect the sidechain motion. The protein and lipids were mixed in the organic phase. For each membrane sample, we dissolved 5–6 mg of peptide in 1.5 ml of trifluoroethanol (TFE) and mixed it with appropriate volumes of POPC, POPE, and cholesterol in 100% chloroform and SM in a chloroform/methanol (50%/50% vol) solution. The resulting ∼4 ml of protein–lipid solution appeared clear. We next removed the organic solvents by flowing nitrogen gas, added 200 μl of cyclohexane to the translucent film, and quickly flash-froze the solution in liquid nitrogen to avoid the separation of protein from lipids. The solution was lyophilized overnight to give a dry homogeneous white powder, which was resuspended in 3 ml of buffer at the desired pH (5.5 or 7.5). The pH 5.5 sample was prepared in a citrate buffer (20 mM citrate, 10 mM NaCl, 2 mM EDTA, 0.2 mM NaN_3_) while the pH 7.5 sample was prepared in a tris buffer (20 mM tris, 10 mM NaCl, 2 mM EDTA, 0.2 mM NaN_3_). This protein–lipid suspension was sonicated in a bath sonicator for 5 s and vortexed for 5 s, and repeated 4–7 times until the solution was homogeneous and translucent. During this process, the pH of the solution was checked with pH paper and adjusted if needed with a few μl of 10 M NaOH or concentrated HCl solutions. This small adjustment never caused the pH of the suspension to exceed 7.6 or fall below 4.5. One-dimensional (1D) ^13^C direct polarization spectra of the proteoliposomes confirm that the membrane is intact (Fig. S1, ESI†).

The protein–lipid suspension was incubated for 60–90 min at room temperature and shaken every 20 minutes to hydrate the proteoliposomes. The suspensions were then subject to 7–11 freeze–thaw cycles between liquid nitrogen temperature and 40 °C to produce large unilamellar vesicles. The pH of the suspension during freeze–thaw remained constant. The homogeneous membrane vesicles were ultracentrifuged at 55 000 rpm at 4 °C for 1.5 hours to obtain a membrane pellet. The pH of the supernatant was verified to be at the desired value. The supernatant was removed and the membrane pellet was washed with 1.5 ml fresh buffer at the same pH to remove residual TFA. The solution was ultracentrifuged again for ∼20 h at 55 000 rpm and 4 °C. The membrane pellet was incubated in a desiccator until it reached a hydration level of ∼40% water by mass relative to the total mass. The membrane pellet was center-packed into 4 mm MAS rotors that contained short borosilicate glass inserters at the bottom. Vespel caps were used to ensure tight fit of the rotors during low-temperature experiments.

### 2.4 Solid-state NMR experiments

Most solid-state NMR experiments were carried out on a 400 MHz (9.4 T) Bruker AVANCE III HD spectrometer using a 4 mm ^1^H/^13^C/^15^N MAS probe or a 4 mm ^1^H/^19^F/^13^C probe. A small number of experiments were conducted on a 600 MHz (14.1 T) Bruker AVANCE III HD spectrometer using a 1.9 mm ^1^H/^19^F/^13^C probe. Magic-angle spinning (MAS) rates were 7, 10 or 10.5 kHz, and experimental temperatures were set to 255 K, 275 K, 295 K and 305–310 K using an FTS refrigeration system. ^13^C, ^15^N, ^1^H, and ^19^F chemical shifts were externally referenced to the adamantane CH_2_ chemical shift at 38.48 ppm on the tetramethylsilane (TMS) scale, the ^15^N-acetylvaline ^15^N signal at 122.0 ppm on the liquid ammonia scale, the POPC Hγ chemical shift 3.26 ppm on the DSS scale, and the 5-^19^F-tryptophan ^19^F signal at −122.1 ppm on the CF_3_Cl scale, respectively. Actual sample temperatures were estimated from the water ^1^H chemical shift using the equation *T*_sample_ (K) = 96.9 × (7.83 − *δ*_H_2_O_).^36^ Typical ^1^H radiofrequency (rf) field strengths were 71 kHz for excitation, 71–83 kHz for decoupling, and 41–55 kHz for ^1^H–^13^C cross-polarization (CP). Typical ^13^C rf field strengths were 45–55 kHz for short pulses and 45.5 kHz for ^1^H–^13^C CP. Typical ^15^N rf field strengths were 35.7 kHz for hard pulses and 33–36 kHz for ^1^H–^15^N CP. Typical ^19^F rf field strengths were 50 kHz for both hard pulses and ^1^H–^19^F CP. ^1^H–^15^N CP conditions were transferred from the conditions optimized for crystalline histidine lyophilized from a pH 6.0 solution.^37^ Typical CP contact times were 1 ms for ^1^H–^13^C CP, 2 ms for ^1^H–^15^N CP and 0.3–0.5 ms for ^1^H–^19^F CP. The CP spin-lock field strength was ramped on the heteronuclear channel, with an amplitude range of 70–100% for ^13^C and 80–100% for ^15^N and ^19^F. Typical recycle delays were 1.7–1.8 s at 255 K and 275 K and 2.0 s at 295 K and 305–310 K.

To resolve the signals of different H19 species in GDR-BM2, we measured 2D ^13^C–^13^C (CC) correlation spectra with 72 ms ^13^C spin diffusion using the CORD sequence.^38^ 2D ^15^N–^13^C (NC) correlation spectra were measured using an out-and-back transferred-echo double-resonance (TEDOR) pulse sequence with a 1.6 ms total REDOR mixing time.^39^ To investigate the H19 sidechain mobility, we measured dipolar-doubled 2D CP ^1^H–^13^C dipolar chemical-shift (DIPSHIFT) correlation spectra. The experiment used the frequency-switched Lee-Goldberg (FSLG) sequence for ^1^H–^1^H homonuclear decoupling.^40,41^ More detailed parameters of the ssNMR experiments are given in Table S1 (ESI†).

### 2.5 Solid-state NMR spectral analysis

All solid-state NMR spectra were processed in the Topspin 4.1.4 software and plotted with custom-written Python scripts using the *nmrglue* package.^42^^19^F spinning sideband patterns were simulated with the solid lineshape analysis tool in the Topspin 4.1.4 software. Resonance assignment of 2D correlation spectra was conducted using the NMRFAM-Sparky software.^43^

To quantify the relative amounts of τ tautomer, π tautomer, and cationic histidine in each peptide, we analyzed the integrated intensities of each histidine species in 2D CC and NC spectra. For each spectrum, the integrated intensities of all peaks belonging to a histidine species were summed and divided by the number of cross peaks to give the average intensity of the histidine state. The percent populations measured from different spectra were averaged to give a consensus value for a histidine species. Uncertainties in the populations were conservatively estimated to be ±5% based on the different values measured from 2D CC and 2D NC spectra. Pearson correlation coefficients were calculated for GDR-BM2, WT-BM2 and WT-AM2 at acidic pH between different histidine and tryptophan states. A total of 12 correlations, due to three histidine states (τ, π and cationic) and four tryptophan states (A, B, C, D), were evaluated.

We simulated DIPSHIFT dephasing curves using a custom-written Python script to obtain the dipolar coupling strengths. We assumed the rigid-limit one-bond C–H dipolar coupling in the imidazole ring to be 23.9 kHz,^21^ multiplied by 2 for dipolar doubling^41^ and by the theoretical scaling factor of 0.577 for the FSLG sequence. Error bars for the normalized intensities *S*/*S*_0_ were propagated from the spectral SNRs using the equation 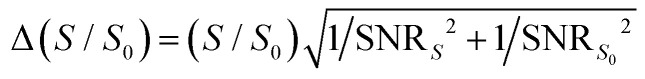. Here, *S* and *S*_0_ are the intensities of the dephased and control spectra, respectively. The uncertainty in the C–H dipolar order parameters (*S*_CH_) was estimated using a Monte Carlo method.^44^ For each dipolar dephasing curve, we randomly perturbed the normalized spectral intensities within the bounds of their respective error bars assuming a normal distribution. The perturbed dipolar dephasing curve was then simulated to obtain an *S*_CH_ value. We iterated this process 1000 times to obtain the standard deviation of the resulting *S*_CH_ values across all iterations. We used the value of two standard deviations (95% confidence interval) as the uncertainty of the order parameter.

## 3. Results and discussion

### 3.1 Proton channel activity of GDR-BM2

To measure proton currents, we expressed full-length GDR-BM2 (1–106), WT-BM2 (1–106), and WT AM2 (1–97) in *Xenopus laevis* oocytes. Fig. 1(c) and (d) show that when the external bathing solution is at pH 5.5 all three proteins display robust inward current. However, when the pH of the bathing solution was switched to 8.5 after the interior of the oocyte has been acidified (low pH_in_), only WT-BM2 shows outward current, while WT-AM2 and GDR-BM2 display zero outward current. Thus, GDR-BM2 is inward rectifying, like WT-AM2. This result is consistent with electrophysiological data of DR-BM2. We also measured current–voltage relationship of WT-BM2, GDR-BM2 and DR-BM2 channels (Fig. 1(e)). GDR-BM2 displays a slightly inward rectified *I*–*V* curve, similar to DR-BM2, while WT-BM2 was slightly outward rectified. The similar functional behavior of GDR-BM2 and DR-BM2 suggests that the S16G mutation does not have a strong effect on the proton conductance.^9^ The recapitulation of the AM2 phenotype by a minimally modified BM2 sequence motivated us to investigate the sidechain structures and interactions of the two key functional residues, H19 and W23, in this BM2 mutant channel.

### 3.2 Protonation and tautomeric structures of H19 from 1D ^13^C and ^15^N NMR spectra

**Fig. 2:**
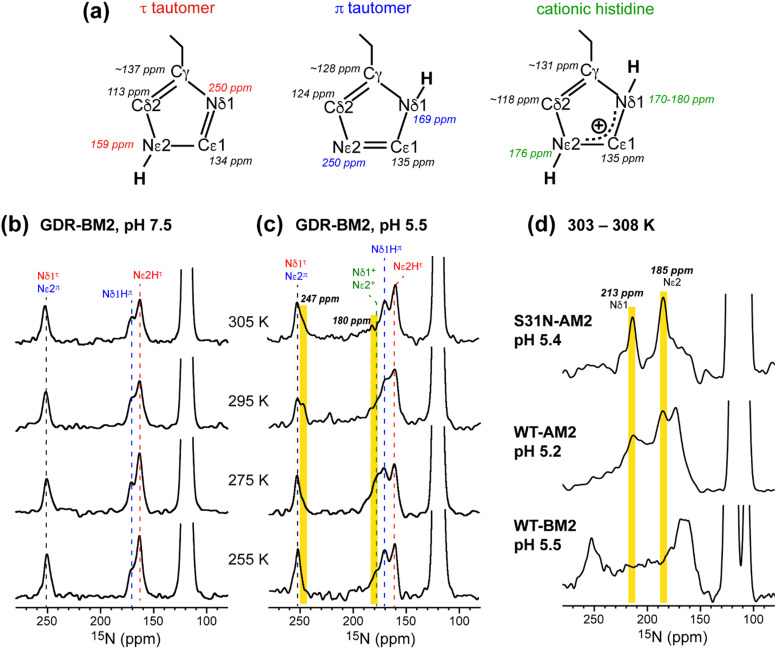
Variable-temperature ^15^N CP spectra of membrane-bound GDR-BM2 at neutral and acidic pH from 255 K to 305 K. (a) Canonical ^13^C and ^15^N chemical shifts of the three histidine structures based on previous studies of M2 proteins^21,45^ and amino acid histidine.^37^ (b) ^15^N spectra of GDR-BM2 at pH 7.5. (c) ^15^N spectra of GDR-BM2 at pH 5.5. (d) Previously reported high-temperature ^15^N spectra of S31N-AM2,^46^ WT-AM2,^22^ and WT-BM2^24^ around pH 5.5. The τ tautomer, π tautomer, and cationic histidine signals are assigned in red, blue, and green, respectively. Yellow shaded bars denote exchange peaks observed at high temperature in the pH 5.5 samples. In all figures, the indicated temperatures of GDR-BM2 are set temperatures for the NMR probe. The estimated sample temperatures are listed in Table S1 (ESI†).

To determine the pH-dependent protonation states and tautomeric structures of H19 in membrane-bound GDR-BM2, we measured 1D ^15^N and ^13^C CP spectra at low temperature. The imidazole ^15^N and ^13^C chemical shifts are exquisitely sensitive to the protonation and tautomeric state of histidine^37^ (Fig. 2(a)). Neutral τ and π tautomers have ^15^N chemical shifts of about 250 ppm for the unprotonated nitrogen and between 160 and 170 ppm for the protonated nitrogen, while cationic histidine has chemical shifts between 170 and 180 ppm for both protonated nitrogen atoms. At 255 K when GDR-BM2 sidechains are immobilized, the aromatic region of the ^15^N spectra show both unprotonated ^15^N intensities at 250 ppm and protonated ^15^N peaks at 163 and 170 ppm (Fig. 2(b) and (c)). These neutral histidine signals are observed at both pH 7.5 and 5.5. In addition, the pH 5.5 spectrum shows intensities between 170 and 180 ppm, corresponding to the cationic histidine.

^13^C CP spectra (Fig. S2, ESI†) are consistent with the ^15^N spectra in reporting the distributions of the histidine species at neutral and acidic pH. Among the three imidazole carbon atoms, the Cδ2 chemical shift is the most diagnostic of the tautomeric and protonation states: Cδ2 resonates at 113–114 ppm for the τ tautomer, 124 ppm for the π tautomer, and 116–119 ppm for cationic histidine. The Cγ chemical shift also varies among the three species, ranging from 137 ppm for the τ tautomer to 128 ppm for the π tautomer (Fig. 2(a)). The ^13^C spectra of H19 (Fig. S2a and b, ESI†) exhibit Cδ2 signals of both τ and π tautomers at pH 7.5 and 5.5, consistent with the ^15^N spectra. In addition, the pH 5.5 spectrum shows intensity at 118 ppm, indicative of cationic histidine. The ^13^C linewidths are broad, indicating that the H19 sidechain structure is disordered, especially for cationic histidine. Natural abundance ^13^C signals of lipids and cholesterol are also observed between 121 ppm and 142 ppm based on the spectrum of the peptide-free VM+ membrane. These lipid signals have low intensities at low temperature and sharpen at high temperature to become resolved from the peptide signals.

**Fig. 3:**
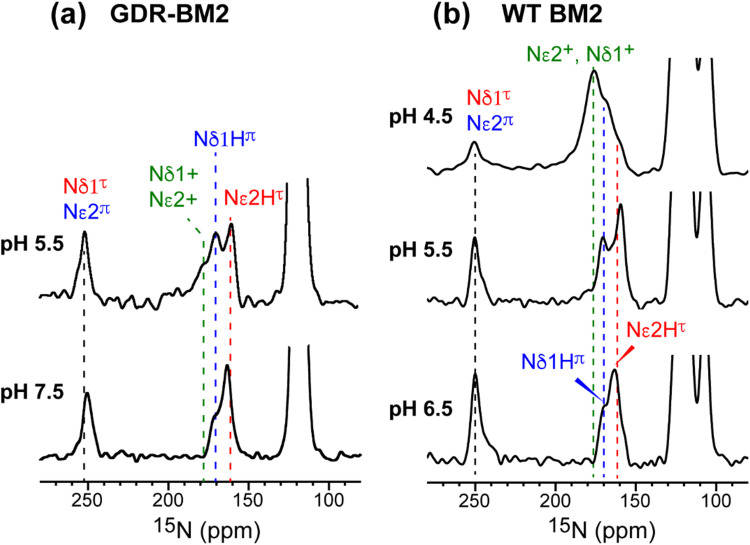
^15^N spectra of membrane-bound GDR-BM2 and WT-BM2 measured at 255 K. (a) Spectra of GDR-BM2 at pH 7.5 and pH 5.5. (b) Previously reported spectra of WT-BM2 at pH 6.5, 5.5 and 4.5.^16^ Between pH 6.5 and 7.5, the two peptides exhibit similar ^15^N chemical shifts and intensity distributions, indicating similar populations of τ and π tautomers. At pH 5.5, GDR-BM2 exhibits higher cationic His intensities than WT-BM2, indicating that the GDR mutant channel stabilizes the cationic histidine. At pH 4.5, WT-BM2 shows predominantly cationic His intensities.

The GDR-BM2 H19 spectra show detailed differences from the spectra of previously studied WT-BM2. At pH 5.5, WT-BM2 H19 lacks the cationic ^15^N signal at 176 ppm (Fig. 3) and the cationic Cδ2 peak at 118 ppm (Fig. S2c, ESI†). Thus, GDR-BM2 preferentially stabilizes cationic histidine over neutral histidine compared to WT-BM2, indicating that the mutant channel has elevated proton dissociation constants (p*K*_a_'s).

### 3.3 Populations of different histidine states in GDR-BM2 from 2D correlation spectra

**Fig. 4:**
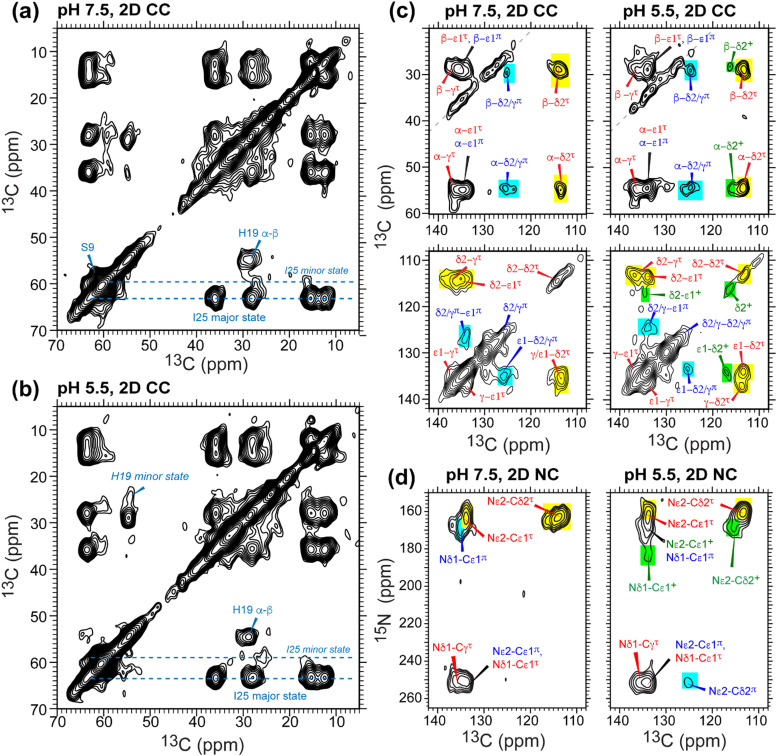
2D ^13^C–^13^C and ^15^N–^13^C correlation spectra of membrane-bound GDR-BM2 to resolve tautomeric and cationic H19 species. (a) 2D CC spectrum at pH 7.5. (b) 2D CC spectrum at pH 5.5. Resonance assignment of the τ tautomer, π tautomer, and cationic His is shown in red, blue and green, respectively. (c) Aromatic region of the 2D CC spectra. (d) Aromatic region of the 2D NC spectra. Colored boxes indicate the integration areas for intensity quantification to obtain the percent populations of τ (yellow), π (cyan) and cationic (green) histidine. The spectra were measured at 255 K.

To further resolve the multiple histidine species and quantify their relative concentrations in GDR-BM2, we measured low-temperature 2D ^13^C–^13^C and ^15^N–^13^C correlation spectra. The Cα and Cβ chemical shifts of S9 and I25 confirm that the protein backbone is α-helical and is pH-independent (Fig. 4(a) and (b)). I25 exhibits two sets of chemical shifts: a high-intensity major conformer has Cα and Cβ chemical shifts of 63 and 36 ppm, indicative of α-helical conformation, and a low-intensity minor conformer with Cα and Cβ chemical shifts of 59 ppm and 35 ppm, consistent with a coil conformation^47^ (Table S2, ESI†). These two sets of signals exist at both pH and maintain the same integrated intensity ratio of about 4 : 1. The origin of the minor I25 conformation is unclear. It may reflect backbone conformational disorder, for example due to a S16G-induced helical kink,^30,31^ or sidechain disorder^48^ due to the location of this residue between W23 and its C-terminal G26D and H27R.

In the aromatic region of the 2D CC spectra, we resolved Cα-aromatic, Cβ-aromatic and aromatic–aromatic correlation peaks (Fig. 4(c)). These signals confirm that neutral histidine sidechains exist at both pH while cationic histidine is present only at pH 5.5. Examples of resolved cationic histidine cross peaks include a Cα–Cδ2 cross peak at (54, 116) ppm and a Cε1–Cδ2 cross peak at (134, 116) ppm. In the 2D NC spectra (Fig. 4(d)), an Nε2–Cδ2 cross peak at (168, 116) ppm and an Nδ1–Cε1 cross peak at (182, 134) ppm also confirm the presence of cationic histidine at pH 5.5. Notably, GDR-BM2 exhibits a single set of τ tautomer and π tautomer chemical shifts, similar to the behavior of WT-AM2 and WT-BM2 but different from H27A-BM2, which displays multiple τ and π conformations.^25^ Due to sensitivity limitations, we do not observe cross peaks between different histidine species. However, previous AM2 studies showed that τ and π tautomers coexist within each channel.^21^ pH titration of many M2 peptides^25,26^ also showed conclusively that multiple charge states (+1, +2 and +3) of tetramers are present within the pH range of 5–7. Thus, the GDR-BM2 channels most likely contain coexisting neutral and cationic histidines within each tetramer.

**Fig. 5:**
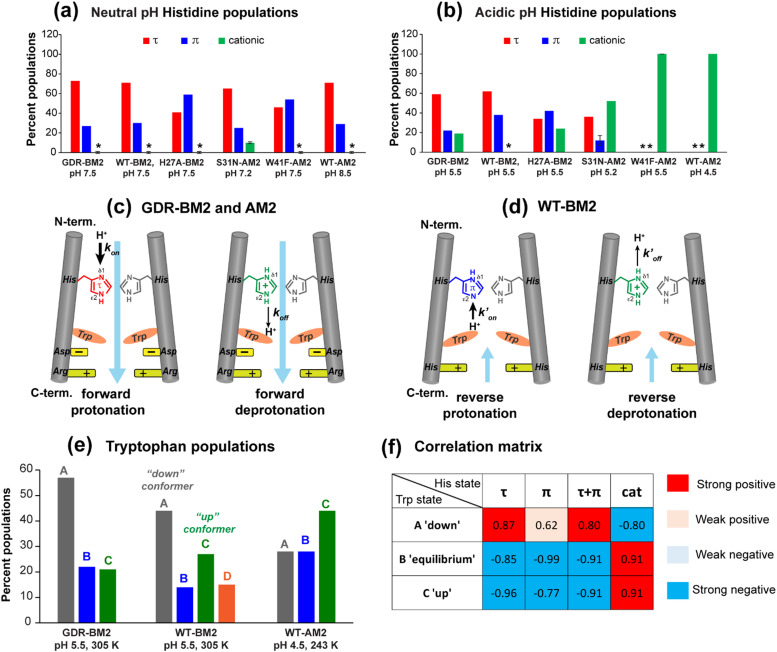
Percent distributions of histidine and tryptophan states for various M2 peptides. (a) Distributions of histidine structures at pH 7.5. (b) Distribution of histidine structures at pH 5.5. These populations are obtained from spectral intensities listed in Table S3 (ESI†). Asterisks indicate 0% populations. (c) Schematic of the main proton transfer processes in inward-rectifying AM2 and GDR-BM2 channels. Only two out of four helices are represented for clarity. The charge state of the histidine on the left is specifically indicated whereas the charge state of the histidine on the right is independent and is shown in grey. The charged residues C-terminal to Trp are Asp and Arg in these channels. Forward deprotonation at acidic pH is facilitated by increased water accessibility around the gating complex. Blue arrow denotes inward proton conduction in these channels. (d) Schematic of the additional proton transfer pathways in WT-BM2. The charged residue C-terminal to Trp is a His. Reverse protonation is facilitated by increased water accessibility around the gating complex. Blue arrow denotes the fact that WT-BM2 conducts protons from the C-terminus to the N-terminus when pH_in_ is acidic. This outward conduction is additional to the inward conduction that is similar to (c). (e) Distributions of Trp conformations obtained from the ^19^F NMR spectra. (f) Pearson correlation matrix between His and Trp states for GDR-BM2, WT-BM2 and WT-AM2 at acidic pH.

To estimate the relative concentrations of the different histidine species in GDR-BM2, we integrated the intensities of the resolved peaks in the 2D spectra and divided them by the number of atoms contributing to each cross peak (Table S3, ESI†). This intensity analysis shows that the τ and π tautomers account for ∼70% and ∼30% of all histidines at pH 7.5 in the mutant channel. At pH 5.5, both tautomer populations decrease while cationic population increases to ∼23% (Fig. 5(a) and (b)). Thus, acidification increased the cationic histidine population without significantly affecting the relative abundance of the τ and π tautomers.

It is noteworthy that the cationic histidine concentrations estimated from the 2D spectra are lower than the values obtained from 1D ^15^N CP spectra. At pH 5.5, the ^15^N spectrum of GDR-BM2 gives an intensity ratio of 2.8 between the protonated NH and the unprotonated nitrogen (N) (Fig. 3(a)). Taking into account the CP efficiency difference of 1.4-fold between NH and N, which was measured on the pH 6.0 amino acid histidine,^22^ the real intensity ratio *I*_NH_/*I*_N_ is about 2.0, indicating a concentration ratio of 2 : 1 for neutral and cationic histidines. Thus, 1D ^15^N spectra indicate a higher cationic histidine content (∼33%) than 2D spectra (∼23%). We attribute this discrepancy to the larger conformational disorder of cationic histidine, which broadens their signals in 2D spectra more than in 1D spectra. Previously measured 2D spectra of WT-BM2 at a cryogenic temperature of 117 K revealed higher cationic histidine intensities compared to spectra measured at 263 K.^24^ Although this intensity difference partly reflects temperature-dependent p*K*_a_'s of the buffer ion the preferential line broadening of cationic histidine compared to neutral histidines due to the conformational disorder of the former is likely a significant contributing factor. In comparison, the two neutral tautomers have similar spectral linewidths, indicating similar structural order. Thus, the τ and π concentration ratios obtained from the spectral intensities are relatively accurate.

To compare the tautomer equilibria of GDR-BM2 with those of previously studied M2 peptides, we integrated the peak intensities of previously reported 1D and 2D CC spectra of WT-BM2,^24^ H27A-BM2,^25^ S31N-AM2,^46^ and W41F-AM2.^26,37^ Table S3 (ESI†) shows that GDR-BM2 resembles S31N-AM2 in having 3-fold larger abundance of the τ tautomer over the π tautomer at pH 5.5, while WT-BM2 has a smaller excess of 2-fold for the τ tautomer. H27A-BM2 departs substantially from the other M2 peptides, with equimolar amounts of τ and π tautomers within experimental uncertainty. In W41F-AM2, the H37 sidechain is predominantly cationic at pH 5.5, without detectable amounts of neutral tautomers. At pH 7.5, this mutant contains more π tautomer than the τ tautomer, similar to H27A-BM2.

The different tautomeric equilibria of the proton-selective histidine in these M2 channels provide important insights into how proton shuttling by histidine is affected by the stability of the channel gate. Below, we give a kinetic model that links the tautomer ratio to the rate constants of histidine protonation and deprotonation. Direct measurements of H37 (*χ*_1_, *χ*_2_) torsion angles in membrane-bound AM2 by ssNMR^21^ and X-ray crystallography^28^ indicated that Nδ1 of neutral histidine points to the N-terminus while Nε2 points to the C-terminus at equilibrium. Neutral histidine does not undergo ring reorientation.^21^ Thus, inward proton conduction from the N-terminus to the C-terminus involves two steps: forward protonation of Nδ1 in the τ tautomer with a rate constant of *k*_on_ to produce cationic histidine (HisH^+^), and forward deprotonation of Nε2 in cationic histidine (*k*_off_) to give a π tautomer (Fig. 5(c)). In addition to these forward processes, reverse protonation (
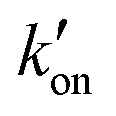
) of Nε2 in the π tautomer and reverse deprotonation of a cationic histidine at Nδ1 (
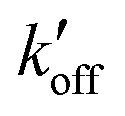
) can occur (Fig. 5(d)). Because proton concentrations are the same on the two sides of the membrane in structural studies, these proton shuttling events by histidine can be described as:
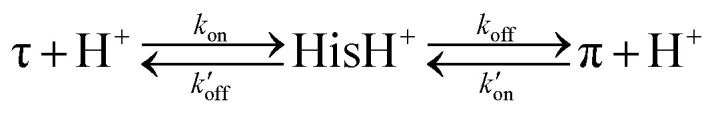


The tautomeric equilibrium constant *K*_tautomer_ can be expressed in terms of the four rate constants through the two underlying proton dissociation equilibria:
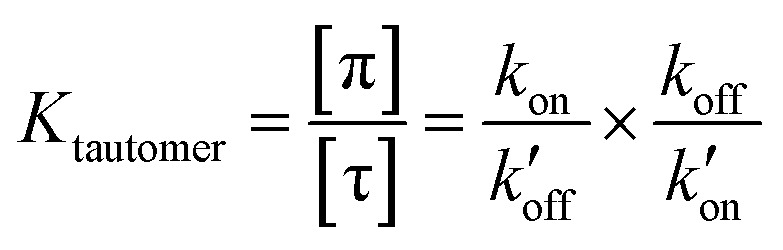


This equation indicates that the relative concentration of the τ and π tautomers at equilibrium is dictated by the rate constants for the forward and reverse protonation and deprotonation events. The free energy barriers associated with these rates depend on the charge state of the histidine tetrad, pH, and conformation of the histidine and the surrounding residues. Multiscale computer simulations gave estimates of the free energy barriers of these protonation and deprotonation events in the gating-competent WT-AM2 channel.^49^ Forward deprotonation (*k*_off_) has the highest energy barrier, followed by reverse protonation (
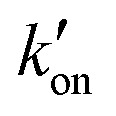
) at acidic pH. These results suggest that the increased [π]/[τ] concentration ratios found in H27A-BM2, WT-BM2 and W41F-AM2 channels result from the increase of the forward deprotonation rate constant *k*_off_ relative to the reverse deprotonation rate constant 
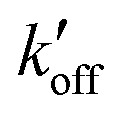
. At acidic pH, an increased [π]/[τ] ratio may alternatively result from the increase of *k*_off_ relative to 
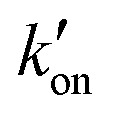
. A larger *k*_off_ requires larger water accessibility of the C-terminal side of histidine. If the gate is tight, then *k*_off_ will decrease, reducing the π tautomer concentration. Therefore, the π tautomer fraction reports on the stability of the Trp gate.

Both S31N-AM2 and WT-AM2 show 3-fold larger τ tautomer concentrations than π tautomer at neutral pH, indicating that reverse deprotonation of Nδ1 is more efficient than forward deprotonation at Nε2, consistent with the presence of a tight Trp gate in these channels due to stabilization by Asp and Arg residues.^17^ In WT-BM2, the [τ]/[π] ratio decreases to ∼2, indicating that forward deprotonation has increased (larger *k*_off_) compared to AM2. This is consistent with the lack of a gate-stabilizing Asp residue in WT-BM2. In GDR-BM2, the [τ]/[π] ratio is similar to that of AM2, indicating that reverse deprotonation at Nδ1 is favored over forward deprotonation at Nε2 in this mutant. Thus, the similar tautomer equilibria of GDR-BM2 and AM2 indicate that the GDR mutations stabilized the gate, explaining the inward rectifying current by this mutant BM2 channel.

The unusually high π tautomer concentration in H27A-BM2 implies that this mutant is similarly efficient in forward deprotonation as reverse deprotonation, even when the H27 removal increased the cationic H19 concentration compared to WT-BM2.^25^ We attribute the equal τ and π concentrations to an especially loose W23 gate, as neither G26 nor A27 can engage in charge interactions with the gate. This loose W23 gate may be chiefly responsible for the polymorphism of the neutral tautomer chemical shifts at neutral pH, which is observed only in H27A-BM2 and not in other M2 peptides. For W41F-AM2, the mutation of W41 to F41 abolished the neutral H37 populations at pH 5.5 and increased the π tautomer concentration at pH 7.5. Thus, the absence of the indole facilitated H37 protonation from the C-terminal side in addition to the N-terminal side.^26^ Taken together, the high populations of the π tautomer in H27A-BM2 and W41F-AM2 are consistent with a loosened gate and the absence of a gate in these two channels.

### 3.4 Proton exchange dynamics and conformational dynamics of H19 in GDR-BM2

While the low-temperature NMR spectra provide information about the relative concentration of the histidine species, high-temperature spectra probe proton transfer dynamics and conformational dynamics of H19. If interconversion among different histidine species occurs at rates that are faster than the chemical shift differences, then chemical shift averaging will be observed. We measured ^15^N CP and ^13^C CP spectra of GDR-BM2 at temperatures from 275 K to 310 K to identify histidine dynamics (Fig. 2 and Fig. S2, ESI†). For the pH 7.5 sample, neither ^15^N nor ^13^C spectra show chemical shift averaging at high temperature, consistent with the lack of proton transfer at neutral pH. In comparison, at pH 5.5, the 1D ^15^N spectrum at 305 K shows an additional signal at 247 ppm (Fig. 2(c)). Since this chemical shift is near the unexchanged chemical shift of 250 ppm for unprotonated nitrogen, this peak can only be attributed to an unequal-population exchange that is skewed predominantly towards neutral histidine. Since both imidazole nitrogen atoms are involved in proton shuttling, a second ^15^N exchange peak is also expected. However, due to the unequal exchange, the second peak is expected to resonate within the NH chemical shift range of 160–180 ppm and thus cannot be resolved from the parent peaks. Nevertheless, we found that the intensity of the 180 ppm peak is significantly lower at 305 K than at 255 K, suggesting that this peak may participate in proton exchange. Variable-temperature ^13^C CP spectra (Fig. S2, ESI†) are qualitatively consistent with the ^15^N spectra. The pH 7.5 spectra show similar intensity envelopes across the temperature range whereas the pH 5.5 spectra show a loss of the cationic Cδ2 intensity at 305 K compared to the low temperature spectra. This reduced intensity again supports the presence of proton exchange, even though narrow exchange peaks are not detected.

The unequal-population proton exchange of H19 in GDR-BM2 differs qualitatively from the proton exchange observed in AM2 peptides (Fig. 2(d)). S31N-AM2 exhibits the fastest exchange among all M2 peptides studied so far, with two narrow ^15^N exchange peaks at 213 ppm and 185 ppm. These chemical shifts are consistent with exchange among two τ tautomers, one π tautomer and one cationic histidine within the same tetrad. WT-AM2 displays the same exchange-averaged ^15^N chemical shifts as S31N-AM2, but have broader linewidths, indicating slower exchange rates. In contrast, the ^15^N spectra of wild-type BM2 at pH 5.5 do not exhibit narrow exchange peaks at high temperature, but show broad intensities between 180 ppm and 220 ppm, suggesting that slow but similar-population exchange may occur between neutral and cationic histidines.

**Fig. 6:**
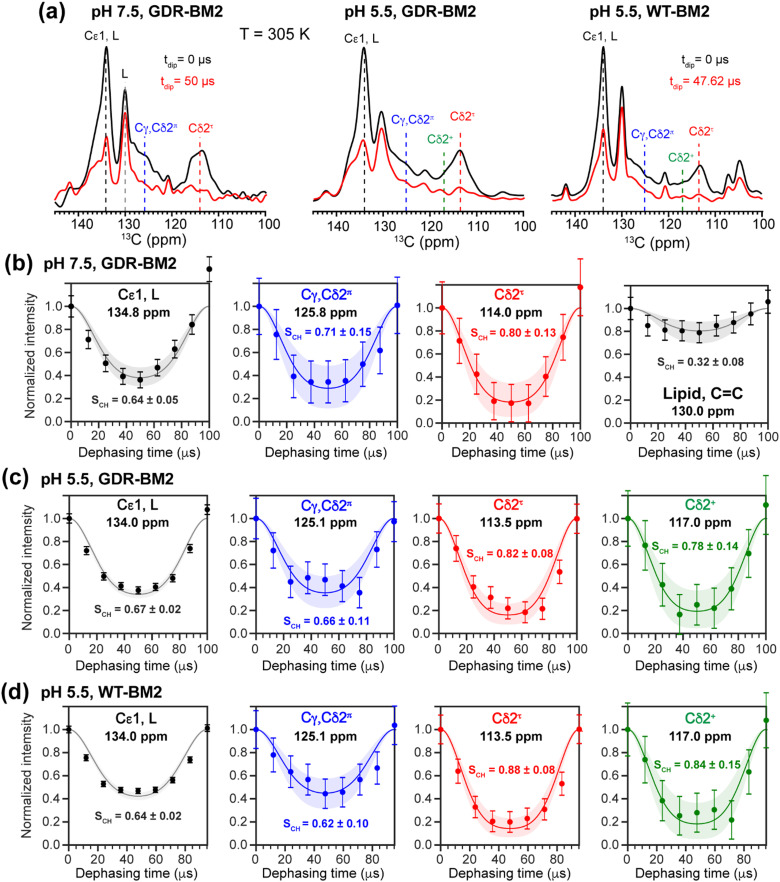
2D ^13^C–^1^H DIPSHIFT data of membrane-bound GDR-BM2 and WT-BM2. The spectra were measured at 305 K under 10 kHz or 10.5 kHz MAS. (a) Aromatic region of the ^13^C dimension of the 2D spectra of the three peptides at C–H dipolar evolution times of 0 μs and half of the rotor period (50 μs or 47.6 μs). (b) C–H dipolar dephasing of GDR-BM2 at pH 7.5. (c) C–H dipolar dephasing of GDR-BM2 at pH 5.5. (d) C–H dipolar dephasing of WT-BM2 at pH 5.5. Error bars were propagated from the spectral signal-to-noise ratios. Best-fit simulations (solid lines) are shown along with uncertainties in the *S*_CH_ values (shaded areas) up to two standard deviations. The rigid-limit C–H dipolar coupling is assumed to be 23.9 kHz.

To investigate whether the H19 sidechains are mobile at high temperature, we measured 2D ^13^C–^1^H DIPSHIFT spectra of GDR-BM2 at pH 7.5 and 5.5 and WT-BM2 spectra at pH 5.5 at 305 K (Fig. 6). In VM+ membranes, the helix bundle backbone is immobilized, allowing the observation of sidechain dynamics. The dipolar dephasing curves of the resolved Cδ2 signals in GDR-BM2 indicate a Cδ2–H order parameter (*S*_CH_) of 0.8–0.9 for the τ tautomer, which is within the range observed for WT-AM2 at pH 4.5 and 8.5 and which suggests partial ring reorientations around the Cβ–Cγ bond.^21^ The π tautomer exhibits a smaller *S*_CH_ of 0.7–0.8, which we attribute to resonance overlap between Cδ2 and the unprotonated Cγ of the π tautomer. These GDR-BM2 order parameters are unaffected by pH, indicating that the H19 sidechain mobility is similar between neutral and acidic pH. Between the WT and mutant BM2, no significant difference is observed in the order parameters, indicating that introduction of D26 and R27 does not significantly alter the H19 mobility.

The Cε1–H bond exhibits a low *S*_CH_ of 0.65 at both pH. This significant reduction of the dipolar coupling suggests that the Cε1–H bond is elongated in GDR-BM2, similar to WT-AM2, which has a *S*_CH_ of 0.72–0.75.^21^ The Cε1–H bond elongation in M2 peptides could result from a combination of chemical exchange with water and cation–π interactions between neighboring histidine rings.

### 3.5 Conformation of W23 in GDR-BM2

**Fig. 7:**
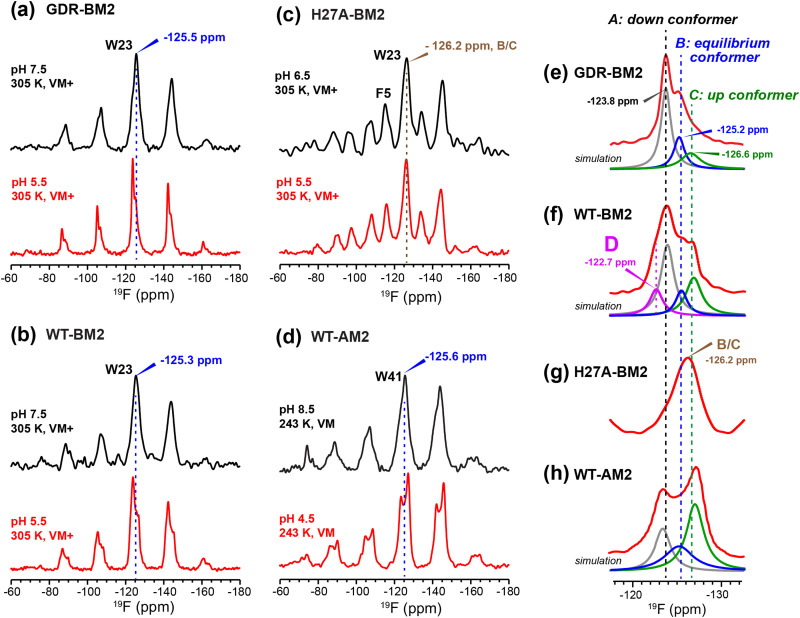
^19^F CP spectra of 5-^19^F-Trp in M2 peptides resolve multiple Trp conformations at acidic pH. For each peptide, two samples were measured, at neutral pH (6.5–8.5) and acidic pH (4.5–5.5). Panels (e)–(h) are zoomed-in centerband regions of the acidic-pH spectra in panels (a)–(d), to better display the partially resolved peaks. Dashed lines guide the eye for the different ^19^F chemical shifts. (a) ^19^F spectra of W23 in GDR-BM2 measured at 305 K. The neutral-pH spectrum shows a single broad peak whereas the acidic-pH spectrum resolves three peaks (A: −123.8 ppm; B: −125.2 ppm; C: −126.6 ppm), as shown in (e). (b) ^19^F spectra of W23 in WT-BM2. The neutral-pH spectrum shows a single broad peak while the acidic-pH spectrum resolves four components (A: −124.1 ppm; B: −125.5 ppm; C: −126.8 ppm; D: −122.7 ppm), as shown in (f). (c) ^19^F spectra of W23 in H27A-BM2. Both the neutral pH and acidic pH spectra show a single broad peak. The latter spectrum's centerband is shown in (g). This peptide also contains 4-^19^F-labeled Phe5 with an isotropic shift of −116 ppm. (d) Low-temperature ^19^F CP spectra of membrane-bound WT-AM2 at pH 8.5 and pH 5.5, adapted from.^16^ The pH 4.5 spectrum resolves three peaks (A: −123.5 ppm; B: −125.4 ppm; C: −127.2 ppm), as shown in (h). Peaks A, B, and C are assigned to a down conformer, an equilibrium conformer, and an up conformer of the indole ring based on an integrated analysis of these spectra and on the structure and dynamics of W41 in AM2.^16^

To relate the H19 sidechain structures and dynamics to the W23 conformation, we next measured ^19^F NMR spectra of 5-^19^F-W23 in GDR-BM2. ^19^F isotropic and anisotropic chemical shifts are sensitive to molecular conformation and dynamics. Previous studies of tryptophan ^19^F NMR spectra in M2 peptides were mostly conducted at low temperature to obtain information about the rigid-limit sidechain conformation.^15,16,25,26,50^ These frozen membranes broadened the signals, preventing the resolution of possible fine features in these ^19^F spectra. To enhance spectral resolution and detect sidechain dynamics, we measured ^19^F CP spectra of 5-^19^F-W23 labeled GDR-BM2, WT-BM2, and H27A-BM2 at a high temperature of 305 K (Fig. 7).^16^ Spinning sideband intensities indicate that all BM2 peptides have ^19^F chemical shift anisotropies (*δ*) of about 45 ppm, near the rigid-limit value, thus the indole sidechain does not undergo large-amplitude motions.

In GDR-BM2, the ^19^F signal is a single broad peak centered at −125.5 ppm at pH 7.5 but partially resolves three signals at −123.8 ppm, −125.2 ppm and −126.6 ppm at pH 5.5 (Fig. 7(a) and (e)). We denote these peaks as state A, B, and C, respectively (Table S4, ESI†). A ^19^F direct polarization spectrum measured with a recycle delay of 5 s at the same temperature shows the same intensity envelope as the ^19^F CP spectrum (Fig. S3, ESI†), indicating that the relative intensities in the CP spectra reflect the abundance of the different Trp conformers in GDR-BM2. The ^19^F NMR spectra of WT-BM2 preserve the same qualitative features as GDR-BM2, with a single broad peak at neutral pH but fine features at acidic pH. Spectral simulations indicate that the low-pH spectrum contains as many as four components, three of which resemble the states A–C seen in GDR-BM2, and a fourth component at −122.7 ppm, which we denote as state D (Fig. 7(b) and (f)). Thus, the W23 gate in GDR-BM2 and WT-BM2 adopts multiple discrete conformations at acidic pH but is more disordered at neutral pH. In contrast, the H27A-BM2 ^19^F spectra show a single broad peak at −126.2 ppm, which is intermediate between the states B and C chemical shifts, at both neutral and acidic pH (Fig. 7(c) and (g)). Finally, the low-temperature ^19^F spectra of W41 in WT-AM2^16^ show a single peak at the state B chemical shift at pH 8.5 but resolves the conformer A and C peaks at pH 4.5 (Fig. 7(d) and (h)).

**Fig. 8:**
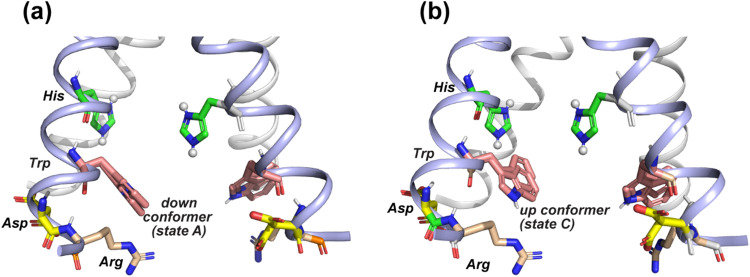
Provisional model of the tryptophan sidechain conformation that regulates the interactions of this residue with the N-terminal proton-selective histidine and the C-terminal charged residues. The crystal structure of AM2 at pH 5.5 (PDB: 4QKM) is used for modeling.^29^ Key residues (H37, W41, D44 and R45) in two neighboring chains (light blue) are shown as sticks. H37 is cationic in this crystal structure. (a) A putative tryptophan down conformer (state A) that interacts with the C-terminal charged residues. The Trp (*χ*_1_, *χ*_2_) torsion angles in the left chain are slightly modified to (−175°, −75°) from the original structure. The original structure has Trp (*χ*_1_, *χ*_2_) torsion angles of (−175°, −90°), which are shown in the chain on the right. (b) A putative tryptophan up conformer (state C) that interacts with the cationic histidine. The Trp (*χ*_1_, *χ*_2_) torsion angles in the left chain are modified to (−155°, +135°) from the original structure to depict this state.

The pH dependence of the states A, B and C intensities among these M2 peptides allows us to assign these ^19^F chemical shifts. State B is dominant for all peptides at neutral pH but decreases in intensity at acidic pH, indicating that this conformation is associated with the neutral state of histidine. Previous measurement of W41 sidechain dynamics in AM2 showed that the indole ring has restricted mobility at neutral pH.^16^ Thus, we attribute conformer B to the equilibrium structure of tryptophan in the absence of cationic histidine. States A and C dominate at acidic pH and decrease in intensities at neutral pH in GDR-BM2, WT-BM2 and WT-AM2, suggesting that these states are associated with indole interactions with the cationic histidine on the N-terminal side and charged residues on the C-terminal side. Importantly, state A is missing in the pH 5.5 spectrum of H27A-BM2, which lacks any charged residues C-terminal to W23. This suggests that state A may be associated with the tryptophan rotamer that interacts with the C-terminal charged residues. In turn, state C must be assigned to the tryptophan rotamer that interacts with the N-terminal cationic histidine. This assignment is in good agreement with the previously reported Gaussian biaxial fluctuations of the W41 indole ring around the Cα–Cβ and Cβ–Cγ bond in WT-AM2.^16^ This motion allows the Trp sidechain to adopt an “up conformer” (state C) that approaches the cationic histidine and a “down conformer” (state A) that approaches the C-terminal charged residues (Fig. 8). In GDR-BM2, state A is predominant over state C at acidic pH, suggesting that W23 interacts more strongly with D26 and R27 than with H19. In H27A-BM2, the W23 ^19^F chemical shift is intermediate between that of the equilibrium state and the up conformer. This is consistent with the lack of C-terminal charged residues to stabilize Trp in this mutant.

State D is observed only in the low-pH WT-BM2 channel, which, unlike other M2 peptides, has a second histidine at H27. The ^19^F chemical shift of state D is close to the chemical shift of the state A, but is even more deshielded than states A, indicating that state D experiences the strongest electron–withdrawing interactions. These observations suggest that state D may correspond to a “down conformer” that engages in simultaneous H19–W23 and H27–W23 cation–π interactions. The absence of state D in GDR-BM2 at pH 5.5, which possesses the mutated R27, might be due to the inability of the arginine sidechain to effectively interact with W23. Crystal structures of WT-AM2 show that R45 forms a salt bridge with D44 of the neighboring helix.^18^ Mutation of this Arg to Cys did not change the inward rectifying proton conduction of WT-AM2, in contrast to Asp mutants.^17^ Thus, an arginine residue at this position might preferentially interact with the aspartate rather than the tryptophan in M2 peptides.

### 3.6 Structure–function relationship of GDR-BM2 and the molecular mechanism of inward rectification

These ssNMR data of H19 and W23 in GDR-BM2 proton channels provide a wealth of new information about the role of the C-terminal TM residues in regulating the M2 channel gate. Low-temperature ^15^N and ^13^C spectra of H19 show that the GDR-BM2 mutant has a higher population of cationic histidine than WT-BM2 at acidic pH. Thus, these mutations slowed down proton dissociation of H19, stabilizing more highly charged tetrads. In addition, the mutations increased the τ tautomer concentration over π tautomers compared to WT-BM2 (Table S3, ESI†), suggesting that reverse deprotonation is more efficient in GDR-BM2 than in WT-BM2. This high [τ] : [π] ratio is similar to the WT-AM2 tautomeric equilibrium.^26^

Although ^19^F NMR spectra of the gating tryptophan have been reported for a number of M2 peptides before, the current study provides the first comprehensive analysis of the multiple conformations of Trp at acidic pH. Interestingly, we resolved three main ^19^F chemical shifts across multiple M2 samples. Conformer B is found at neutral pH for all M2 channels, thus it represents the equilibrium conformation of Trp in a predominantly neutral channel. At acidic pH, we observed varying intensities of conformers A and C in most M2 peptides except for H27A-BM2. Comparison with the previously studied W41 dynamics in WT-AM2 suggests that these two states likely arise from Trp interactions with the C-terminal charged residues (down conformer) and with the N-terminal cationic histidine (up conformer). The down conformer A is stabilized by charge interaction with aspartate, arginine or H27, while the up conformer C is stabilized by interactions with the proton-selective histidine (Fig. 8).

To provide further evidence of the correlation between the histidine charge state and tryptophan conformers, we calculated the Pearson's correlation coefficients between His and Trp populations for GDR-BM2, WT-BM2 and WT-AM2 at acidic pH (Fig. 5(f)). The correlation matrix shows that cationic histidine is strongly positively correlated with the up conformer C of tryptophan and negatively correlated with the down conformer A. In comparison, neutral histidine is strongly positively correlated with the down conformer A and negatively correlated with the up conformer C. These results thus support the model that conformer C interacts with cationic histidine on its N-terminal side whereas conformer A occurs in the presence of neutral histidine and likely interacts with the C-terminal charged residues. The only apparent discrepancy is found for WT-BM2 at pH 5.5, which does not show a detectable amount of cationic histidine at moderate temperature (263 K) but exhibits the Trp up conformer. However, at cryogenic temperature, 2D spectra of WT-BM2 revealed a significant amount of cationic histidine signals,^24^ suggesting that the imidazolium rings are dynamically disordered in this channel at moderate temperature. Thus, the presence of the Trp up conformer C in WT-BM2 is still consistent with interactions with cationic histidine.

The origin of state D in WT-BM2 is not entirely clear. We hypothesize that it may arise from simultaneous interactions of W23 with cationic H19 and H27 at acidic pH. GDR-BM2 at pH 5.5 displays a lower population of the up conformer C compared to WT-AM2 at pH 4.5. A possible reason for this difference is that W23 interaction with D26 and R27 in the mutant might destabilize the W23–H19 interaction. The strong interaction of W23 with the Asp and Arg in the mutant, which is manifested by the high down-conformer A peak (Fig. 7(e)), could also be contributed by the backbone conformation of the mutant, which may differ from the WT-BM2. Regardless of the exact structural reasons, the low population of the W23 up conformer in GDR-BM2 is correlated with the unequal-population proton exchange of H19. This suggests that insufficient H19–W23 cation–π interactions might contribute to the inhibition of proton dissociation of cationic histidine.

The chemical and conformational structures of H19 and W23 observed here in GDR-BM2 provide important insights into the gating mechanism of M2 channels. GDR-BM2 exhibits higher populations of cationic and τ tautomeric histidines compared to WT-BM2. These imply that the tryptophan gate in the mutant restricts C-terminal proton dissociation to increase the cationic histidine content and accelerates the reverse deprotonation to increase the τ tautomer population. Therefore, these data support the model that inward rectification in M2 channels is caused by a strong structural asymmetry among the aromatic and charged residues that combines to slow forward deprotonation and speed up reverse deprotonation. Although this proton blockade on the C-terminal side might seem counter-productive for proton conduction, increased tetrad charge by retaining cationic histidines ultimately leads to a global conformational change of the four-helix bundle,^29,51,52^ which dilates the C-terminal pore to rapidly increase proton conduction.

## 4. Conclusions

Mutations of three residues in BM2 to the corresponding AM2 residues generated an inward rectifying BM2 channel. The GDR-BM2 channel has similar proton conduction behavior as the previously studied DR-BM2 mutant, suggesting that the structural characteristics observed here may primarily arise from the G26D and H27R mutations C-terminal to the Trp residue. Comparison of the GDR-BM2 spectra with previously measured spectra of other M2 peptides provided comprehensive insights into the influence of the C-terminal charged residues on the W23 conformation, which in turn regulate the histidine protonation and tautomeric equilibria. We find that inward rectifying proton currents are associated with increased populations of cationic and τ tautomer histidine. At acidic pH, the tryptophan residue adopts several ordered conformations, which most likely represent a down conformation that interacts with the C-terminal TM residues and an up conformation that interacts with the N-terminal proton-selective histidine. Hence, gating in influenza M2 channels is achieved by a constellation of residues rather than by a single tryptophan. These results illustrate how a small number of charged and aromatic residues, when appropriately positioned in space, can form dynamic and multifaceted interactions to regulate proton conduction by this minimalist proton channel.

## Author contributions

Y. P. measured the solid-state NMR spectra. M. M. synthesized the isotopically labeled GDR-BM2(1–33) peptide. C. M., H. T. and J. W. measured proton currents of BM2 and AM2 samples. Y. P. and M. H. analyzed and interpreted the NMR spectra. All authors contributed to data interpretation. Y. P. and M. H. wrote the paper with input from all authors. All authors approved the final version of the manuscript.

## Data availability

The data supporting this article have been included as part of the ESI.†

## Conflicts of interest

There are no conflicts to declare.

## Supplementary Material

CP-026-D4CP01648C-s001
