# Supplementary material for: Structure and dynamics of the proton-selective histidine and the gating tryptophan in an inward rectifying hybrid influenza B and A virus M2 proton channel
Source: Phys Chem Chem Phys. 2024 Jul 16;26(30):20629–44. doi: 10.1039/d4cp01648c (PMC11290064; doi:10.1039/d4cp01648c)
Supplement: CP-026-D4CP01648C-s001 [file CP-026-D4CP01648C-s001.pdf]

Electronic Supplementary Material (ESI) for Physical Chemistry Chemical Physics.
This journal is © the Owner Societies 2024

Supporting Information

Structure and Dynamics of the Proton-Selective Histidine and the Gating Tryptophan in an Inward Rectifying Hybrid Influenza B and A Virus M2 Proton Channel

Yanina Pankratova [1], Matthew J. McKay [1], Chunlong Ma [2], Haozhou Tan [3], Jun Wang [3], and Mei Hong [1*]

[1] Department of Chemistry, Massachusetts Institute of Technology, 170 Albany Street, Cambridge, MA 02139

[2] Department of Pharmacology and Toxicology, College of Pharmacy, University of Arizona, Tucson, Arizona, 85721, United States

[3] Department of Medicinal Chemistry, Rutgers University, 160 Frelinghuysen Road, Piscataway, NJ 08854

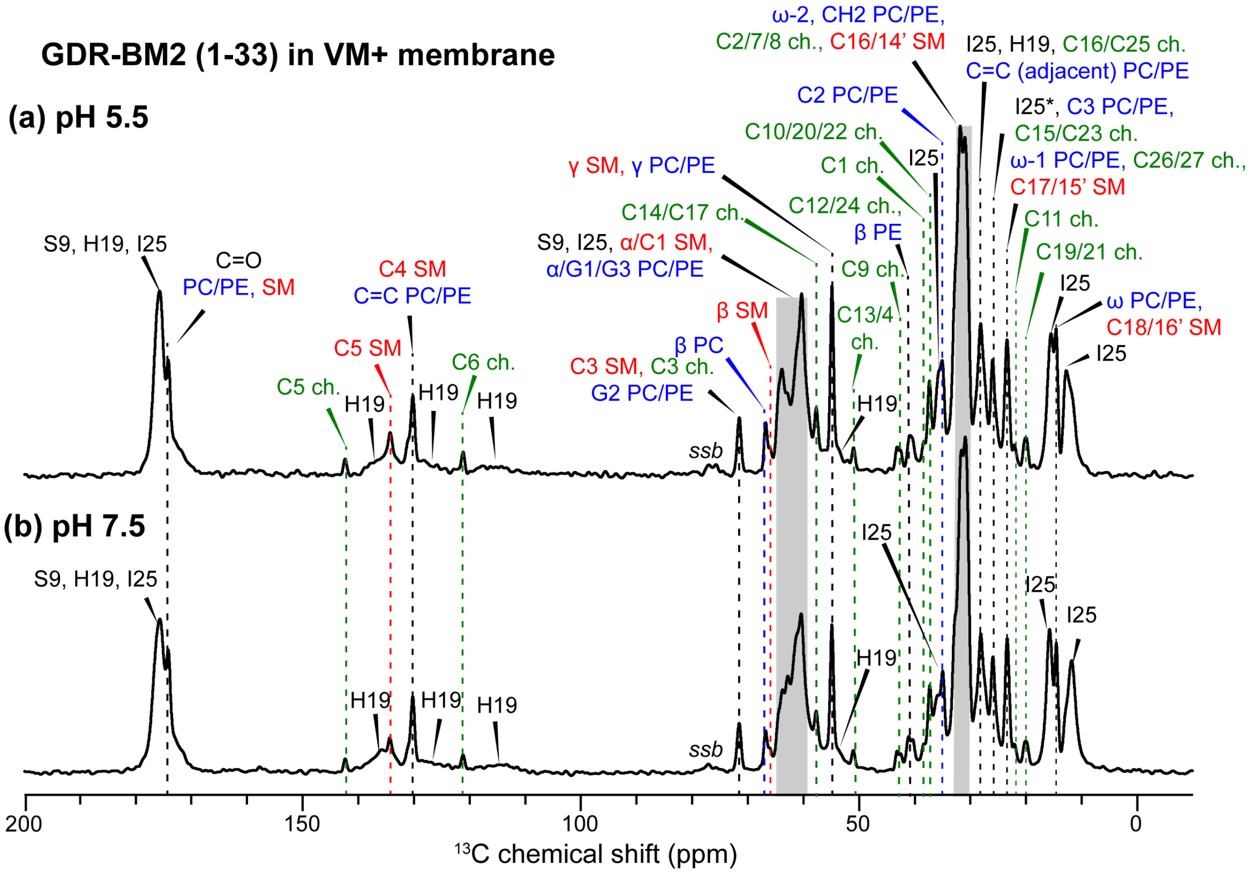

Figure S1. 1D ^{13}C direct polarization spectra of GDR-BM2 (1-33) in VM+ membranes at pH 5.5 (**a**) and pH 7.5 (**b**). These spectra were measured under 10 kHz MAS at 305 K on a 400 MHz NMR. Assignments are shown in blue for POPC and POPE peaks, red for sphingomyelin (SM) peaks and green for cholesterol (ch) peaks. These lipid chemical shifts are standard, thus ruling out hydrolysis in these membranes. "*ssb*" denotes spinning sidebands. Shaded areas denote overlapped peptide and lipid ^{13}C signals.

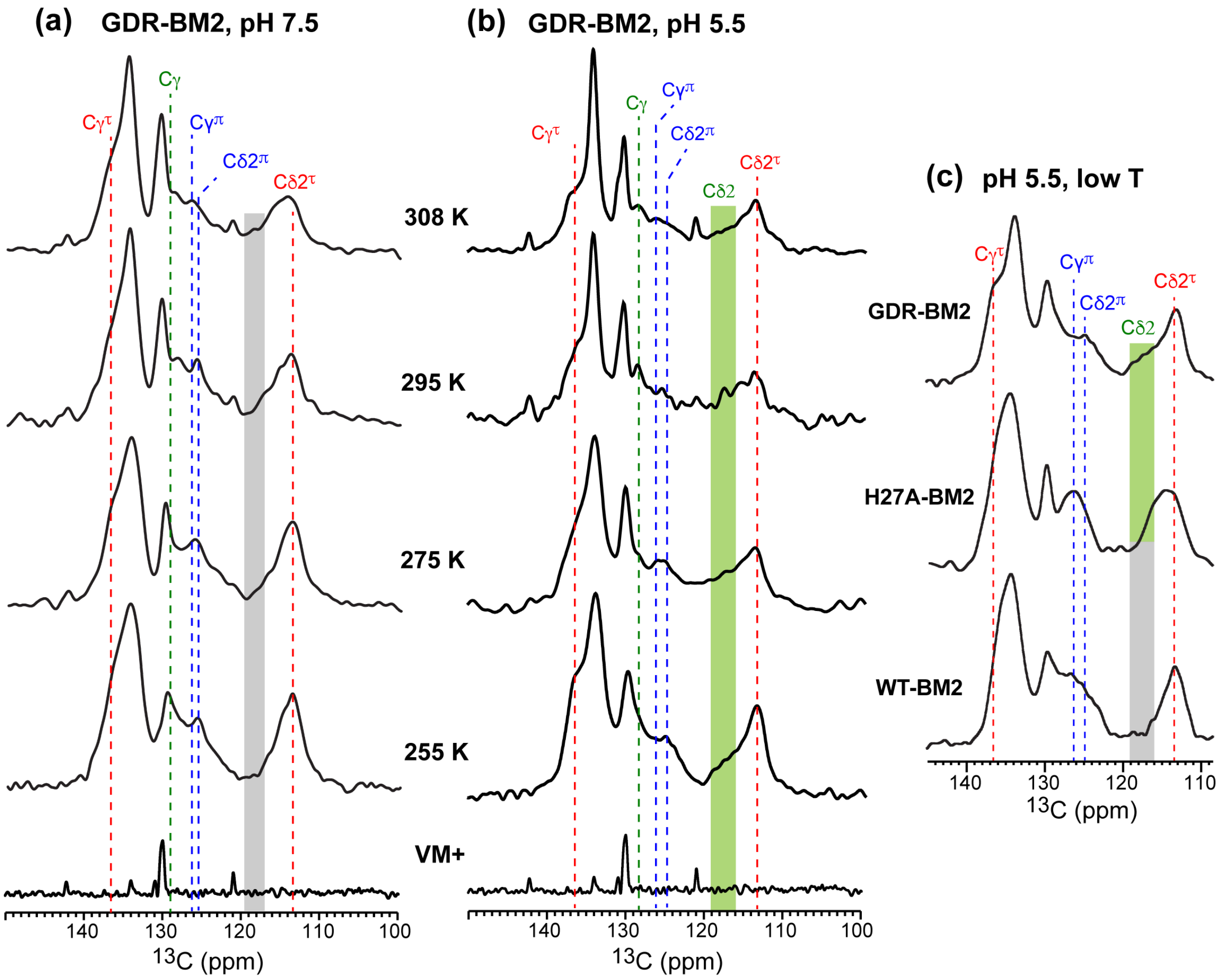

Figure S2. **Histidine imidazole region of the ^{13}C CP spectra of membrane-bound GDR-BM2 and other M2 peptides**. Dashed lines guide the eye for the chemical shifts of different tautomeric and charged histidines. (**a**) ^{13}C spectra of GDR-BM2 at pH 7.5 from 255 K to 308 K (probe set temperatures). No cationic H19 Cδ2 intensities are observed (gray band). The spectrum of the peptide-free VM+ membrane is shown at the bottom to indicate the natural abundance lipid ^{13}C chemical shifts. (**b**) ^{13}C spectra of GDR-BM2 at pH 5.5 from 255 K to 308 K. Cationic Cδ2 intensities (green band) are observed at low temperature and broaden at high temperature. (**c**) Comparison of the low-temperature ^{13}C spectra of membrane-bound GDR-BM2 with previously measured WT-BM2 [1] and H27A-BM2 [2] spectra at pH 5.5. Cationic Cδ2 intensities are observed in GDR-BM2 but not in WT-BM2 and only weakly in H27A-BM2.

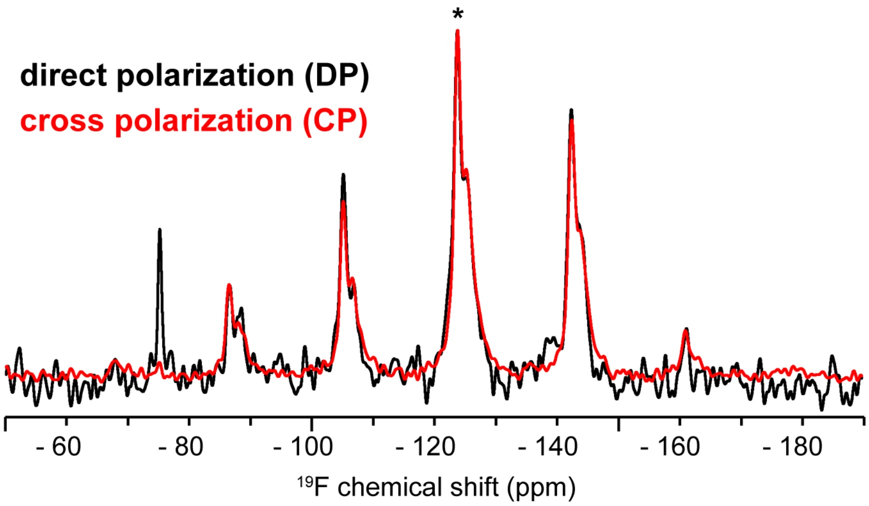

Figure S3. ^{19}F CP and DP spectra of GDR-BM2 at pH 5.5, measured at 308 K under 7 kHz MAS. The DP spectrum (black) was measured with a 5 s recycle delay whereas the CP spectrum (red) was measured with a ^{1}H-^{19}F CP contact time of 350 μs. The two spectra overlap well, indicating that the intensity ratios obtained from the CP spectra reflect the relative abundance of the different Trp species in the peptide. Due to different numbers of scans, the DP spectrum was scaled 14.68 times to match the -123.8 ppm intensity (indicated with an asterisk) in the CP spectrum.

Table S1. Detailed parameters for the solid-state NMR experiments.

Experiment	NMR parameters	Expt. time
	**GDR-BM2 (1-33), pH 7.5, VM$^+$**	
1D ^{13}C DP<br>Figure S1	B_0 = 400 MHz (9.4 T), MAS 10 kHz;<br>**T_{set} = 305 K**, water ^{1}H 4.71 ppm, T_{sample} = + 29^{0}C, ns = 12k, d1 = 5s<br>^{13}C excitation 52.6 kHz, ^{1}H TPPM decoupling 68 kHz	17 h
1D ^{13}C CP<br>Figure S2a	B_0 = 400 MHz (9.4 T), MAS 10 kHz;<br>^{1}H excitation 71.43 kHz, ^{1}H TPPM decoupling 78 kHz; $\tau_{HC\ CP}$ = 1 ms; 1H CP 55.45 kHz, ^{13}C CP 45.45 kHz, ramp on ^{13}C 70-100%;<br>**T_{set} = 255 K:** water ^{1}H 5.26 ppm, T_{sample} = -24^{0}C, ns=4k, d1=1.7 s<br>**T_{set} = 275 K:** water ^{1}H 5.04 ppm, T_{sample} = -2.6^{0}C, ns=6.25k, d1=1.7 s<br>**T_{set} = 295 K:** water ^{1}H 4.79 ppm, T_{sample} = +22^{0}C, ns=5k, d1=2 s<br>**T_{set} = 310 K:** water ^{1}H 4.65 ppm, T_{sample} = +35^{0}C, ns=20k, d1=2 s	 <br><br>1.9 h<br>3.0 h<br>2.8 h<br>11.4 h
1D ^{15}N CP<br>Figure 2b, 3a	B_0 = 400 MHz (9.4 T), MAS 10 kHz;<br>^{1}H excitation 71.43 kHz, ^{1}H TPPM decoupling 71.43 kHz; $\tau_{HN\ CP}$ = 2 ms ^{1}H CP 41 kHz, ^{15}N CP 33.3 kHz, ramp on ^{15}N 80-100%;<br>**T_{set} = 255 K:** water ^{1}H 5.26 ppm, T_{sample} = -24^{0}C, ns=40k, d1=1.7 s<br>**T_{set} = 275 K:** water ^{1}H 5.04 ppm, T_{sample} = -2.6^{0}C, ns=61k, d1=1.7 s<br>**T_{set} = 295 K:** water ^{1}H 4.79 ppm, T_{sample} = +22^{0}C, ns=67k, d1=2 s<br>**T_{set} = 305 K:** water ^{1}H 4.64 ppm, T_{sample} = +36^{0}C, ns=80k, d1=2 s	 <br><br>19.3 h<br>29.5 h<br>38.1 h<br>45.5 h
2D ^{13}C-^{13}C<br>CORD<br>Figure 4a, 4c	B_0 = 400 MHz (9.4 T), MAS 10 kHz;<br>**T_{set} = 255 K:** water ^{1}H 5.26 ppm, T_{sample} = -24^{0}C, ^{1}H excitation 71.43 kHz, ^{1}H TPPM decoupling 78 kHz; $\tau_{HC\ CP}$ = 1 ms; ^{1}H CP 55.45 kHz, ^{13}C CP 45.45 kHz, ramp on ^{13}C 70-100%; τ_{CORD} = 72.0 ms, t_1 = 3 ms (TD2 = 120), t_2 = 18 ms, ns=576, d1=1.7 s	32.6 h
2D ^{15}N-^{13}C<br>TEDOR<br>Figure 4d	B_0 = 400 MHz (9.4 T), MAS 10 kHz;<br>**T_{set} = 255 K:** water ^{1}H 5.26 ppm, T_{sample} = -24^{0}C, ^{1}H excitation 71.43 kHz, ^{1}H TPPM decoupling 78 kHz; $\tau_{HC\ CP}$ = 1 ms; 1H CP 55.45 kHz, ^{13}C CP 45.45 kHz, ramp on ^{13}C 70-100%; ^{15}N recoupling @ 35.7 kHz<br>T_{REDOR} = 1.6 ms, t_1 = 2.5 ms (TD2 = 50) or t_1 =5 ms (TD2 = 100), t_2 = 18 ms, ns=1152, d1=1.8 s	38.4 h
2D ^{13}C-^{1}H<br>doubled<br>DIPSHIFT<br>Figure 6a, 6b	B_0 = 400 MHz (9.4 T), MAS 10 kHz;<br>**T_{set} = 305 K:** water ^{1}H 4.64 ppm, T_{sample} = +36^{0}C, ns=8k, d1=2 s;<br>^{1}H excitation 71.43 kHz, ^{1}H TPPM decoupling 78 kHz; $\tau_{HC\ CP}$ = 1 ms; ^{1}H CP 55.45 kHz, ^{13}C CP 45.45 kHz, ramp on ^{13}C 70-100%; FSLG decoupling 71.43 kHz, ^{13}C π-pulse 45.45 kHz; 9 points	41.0 h
1D ^{19}F CP<br>Figure 7a	B_0 = 400 MHz (9.4 T), MAS 7 kHz;<br>**T_{set} = 305 K:** water ^{1}H 4.73 ppm, T_{sample} = +27^{0}C, ns=30k, d1=2 s; ^{1}H excitation 71.43 kHz, ^{1}H TPPM decoupling 71.43 kHz; $\tau_{HF\ CP}$ = 0.3 ms; ^{1}H CP 50 kHz, ^{19}F CP 50 kHz, ramp on ^{19}F 80-100%	17.1 h
	**GDR-BM2 (1-33), pH 5.5, VM$^+$**	

1D ^{13}C DP<br>Figure S1	B_0 = 400 MHz (9.4 T), MAS 10 kHz;<br>**T_{set} = 305 K**, water ^{1}H 4.71 ppm, T_{sample} = +29 ^{0}C, ns = 10k, d1 = 5s<br>^{13}C excitation 55.6 kHz, ^{1}H TPPM decoupling 68 kHz	14.25<br>h
1D ^{13}C CP<br>Figure S2b	B_0 = 400 MHz (9.4 T), MAS 10 kHz;<br>^{1}H excitation 71.43 kHz, ^{1}H decoupling 78 kHz; $\tau_{HC\ CP}$ = 1 ms; ^{1}H CP 55.45 kHz, ^{13}C CP 45.45 kHz, ramp on ^{13}C 70-100%;<br>**T_{set} = 255 K:** water ^{1}H 5.21 ppm, T_{sample} = -19^{0}C, ns=4k, d1=1.7 s<br>**T_{set} = 275 K:** water ^{1}H 5.01 ppm, T_{sample} = 0^{0}C, ns=2k, d1=1.7 s<br>**T_{set} = 295 K:** water ^{1}H 4.77 ppm, T_{sample} = +24^{0}C, ns=2k, d1=2 s<br>**T_{set} = 308 K:** water ^{1}H 4.65 ppm, T_{sample} = +35^{0}C, ns=15k, d1=2 s	 <br><br>1.9 h<br>1.0 h<br>1.1 h<br>8.5 h
1D ^{15}N CP<br>Figure 2c, 3a	B_0 = 400 MHz (9.4 T), MAS 10 kHz;<br>^{1}H excitation 71.43 kHz, ^{1}H decoupling 71.43 kHz; $\tau_{HN\ CP}$ = 2 ms, ^{1}H CP 41 kHz, ^{15}N CP 33.3 kHz, ramp on ^{15}N 80-100%;<br>**T_{set} = 255 K**: water ^{1}H 5.21 ppm, T_{sample} = -19^{0}C; ns=81k, d1=1.7 s<br>**T_{set} = 275 K**: water ^{1}H 5.02 ppm, T_{sample} = -1^{0}C; ns=116k, d1=1.7 s<br>**T_{set} = 295 K**: water ^{1}H 4.76 ppm, T_{sample} = -24^{0}C; ns=54k, d1=2 s<br>**T_{set} = 305 K**: water ^{1}H 4.66 ppm, T_{sample} = +34^{0}C; ns=122k, d1=2 s	 <br><br>39.2 h<br>56.1 h<br>30.7 h<br>69.4 h
2D ^{13}C-^{13}C<br>CORD<br>Figure 4b, 4c	B_0 = 400 MHz (9.4 T), MAS 10 kHz;<br>**T_{set} = 255 K:** water ^{1}H 5.21 ppm, T_{sample} = -19^{0}C, ^{1}H excitation 71.43 kHz, ^{1}H TPPM decoupling 78 kHz; $\tau_{HC\ CP}$ = 1 ms; 1H CP 55.45 kHz, ^{13}C CP 45.45 kHz, ramp on ^{13}C 70-100%; τ_{CORD} = 72.0 ms, t_1 = 3 ms (TD2 = 120), t_2 = 18 ms, ns=608, d1=1.7 s	34.5 h
2D ^{15}N-^{13}C<br>TEDOR<br>Figure 4d	B_0 = 400 MHz (9.4 T), MAS 10 kHz;<br>**T_{set} = 255 K:** water ^{1}H 5.21 ppm, T_{sample} = -19^{0}C, ^{1}H excitation 71.43 kHz, ^{1}H TPPM decoupling 78 kHz; $\tau_{HC\ CP}$ = 1 ms; 1H CP 55.45 kHz, ^{13}C CP 45.45 kHz, ramp on ^{13}C 70-100%; ^{15}N recoupling @ 35.7 kHz<br>T_{REDOR} = 1.6 ms, t_1 = 2.5 ms (TD2 = 50), t_2 = 18 ms, ns=1632, d1=1.8 s	40.8 h
2D ^{13}C-^{1}H<br>doubled<br>DIPSHIFT<br>Figure 6a, 6c	B_0 = 400 MHz (9.4 T), MAS 10 kHz;<br>**T_{set} = 305 K:** water ^{1}H 4.66 ppm, T_{sample} = +34^{0}C, ns=4k, d1=2 s;<br>^{1}H excitation 71.43 kHz, ^{1}H TPPM decoupling 78 kHz; $\tau_{HC\ CP}$ = 1 ms; 1H CP 55.45 kHz, ^{13}C CP 45.45 kHz, ramp on ^{13}C 70-100%; FSLG decoupling 71.43 kHz, ^{13}C π-pulse 45.45 kHz; 9 points	20.5 h
1D ^{19}F CP<br>Figure 7a, 7e,<br>S2	B_0 = 400 MHz (9.4 T), MAS 7 kHz;<br>**T_{set} = 308 K:** water ^{1}H 4.71 ppm, T_{sample} = +29^{0}C, ns=15k, d1=2 s;<br>^{1}H excitation 71.43 kHz, ^{1}H TPPM decoupling 71.43 kHz; $\tau_{HF\ CP}$ = 0.3 ms; 1H CP 50 kHz, ^{19}F CP 50 kHz, ramp on ^{19}F 80-100%	8.5 h
1D ^{19}F DP<br>Figure S3	B_0 = 400 MHz (9.4 T), MAS 7 kHz;<br>**T_{set} = 308 K:** water ^{1}H 4.71 ppm, T_{sample} = +29^{0}C, ns=1k, d1=5 s;<br>^{1}H TPPM decoupling 71.43 kHz;^{19}F excitation 50 kHz	1.4 h
	**WT-BM2 (1-33), pH 7.5, VM^{+}**	
1D ^{19}F CP<br>Figure 7b	B_0 = 400 MHz (9.4 T), MAS 7 kHz;<br>**T_{set} = 308 K:** water ^{1}H 4.71 ppm, T_{sample} = + 30 ^{0}C, ns=165 k, d1=2 s;<br>^{1}H excitation 71.43 kHz, ^{1}H TPPM decoupling 62.5 kHz; $\tau_{HF\ CP}$ = 0.5 ms; 1H CP 34.5 kHz, ^{19}F CP 52 kHz, ramp on ^{19}F 80-100%	93.8 h

	**WT-BM2 (1-33), pH 5.5, VM$^+$**	
2D ^{13}C-^{1}H doubled DIPSHIFT Figure 6a, 6d	B_0 = 600 MHz (14.1 T), MAS 10.5 kHz;<br>**T_{set} = 305 K:** water ^{1}H 4.73 ppm, T_{sample} = +27 ^{0}C, ns= 12.5 k, d1=2 s;<br>^{1}H excitation 71.43 kHz, ^{1}H TPPM decoupling 71.43 kHz; $\tau_{HC\ CP}$ = 1 ms; 1H CP 61.7 kHz, ^{13}C CP 61.1 kHz, ramp on ^{13}C 70-100%; FSLG decoupling 71.43 kHz, ^{13}C π-pulse 62.5 kHz; 9 points	64 h
1D ^{19}F CP Figure 7b, 7f	B_0 = 600 MHz (14.1 T), MAS 10.5 kHz;<br>**T_{set} = 305 K:** water ^{1}H 4.73 ppm, T_{sample} = +27 ^{0}C, ns=25k, d1=2 s;<br>^{1}H excitation 71.43 kHz, ^{1}H TPPM decoupling 71.43 kHz; $\tau_{HF\ CP}$ = 0.3 ms; 1H CP 65.4 kHz, ^{19}F CP 71.43 kHz, ramp on ^{19}F 70-100%	14.2 h
	**H27A-BM2 (1-33), pH 6.5, VM$^+$**	
1D ^{19}F CP Figure 7c	B_0 = 400 MHz (9.4 T), MAS 7 kHz;<br>**T_{set} = 308 K:** water ^{1}H 4.74 ppm, T_{sample} = +26 ^{0}C, ns=20 k, d1=2 s;<br>^{1}H excitation 71.43 kHz, ^{1}H TPPM decoupling 71.43 kHz; $\tau_{HF\ CP}$ = 0.35 ms; 1H CP 34 kHz, ^{19}F CP 50 kHz, ramp on ^{19}F 90-100%	11.4 h
	**H27A-BM2 (1-33), pH 5.5, VM$^+$**	
1D ^{19}F CP Figure 7g	B_0 = 600 MHz (14.1 T), MAS 10.5 kHz;<br>**T_{set} = 305 K:** water ^{1}H 4.72 ppm, T_{sample} = +28 ^{0}C, ns= 15k, d1=2 s;<br>^{1}H excitation 71.43 kHz, ^{1}H TPPM decoupling 71.43 kHz; $\tau_{HF\ CP}$ = 0.5 ms; 1H CP 50 kHz, ^{19}F CP 58.8 kHz, ramp on ^{19}F 70-100%	8.5 h

Table S2. ^{13}C and ^{15}N chemical shifts of membrane-bound GDR-BM2 at pH 7.5 and pH 5.5. Minor conformations (indicated by an asterisk) are observed for I25 and H19.

Residue	pH	N	C	Cα	Cβ	Cγ1	Cγ2	Cδ1	Cγ	Cδ2	Cε1	Nδ1	Nε2
S9	7.5	117.7	-	59.8	-	-	-	-	-	-	-	-	-
	5.5	116.6	-	59.4	61.6	-	-	-	-	-	-	-	-
I25	7.5	122	-	63.2	36.3	28.0	15.5	12.5	-	-	-	-	-
	5.5	121.3	-	63.6	36.1	28.0	15.4	12.5	-	-	-	-	-
I25 *	7.5			59.3	34.8	27.7	16.0	11.7					
	5.5			59.8	35.4	26.0	15.8						
H19 τ	7.5	120.2	175.1	55.0	28.6	-	-	-	137.4	114.0	134.7	**250.8**	163.4
H19 π		120.2	174.8	54.8	28.8	-	-	-	-	125.8	134.9	168.9	**250.4**
H19 +		-	-	-	-	-	-	-	-	-	-	-	-
H19 τ	5.5	118.7	175.5	54.7	29.0	-	-	-	136.5	113.3	134.0	**250.5**	159.6
H19 π		118.7	175.2	54.6	29.1	-	-	-	-	125.1	134.0	167.3	**250.3**
H19 +		-	174.3	54.38	28.28	-	-	-	-	116.5	134.2	182.1	167.9
H19 *	5.5	-	-	54.4	25.1	-	-	-	136.2	-	-	-	-

Table S3. Percent populations of τ tautomer, π tautomer, and cationic histidine for the proton-selective H19 in BM2 and the proton-selective H37 in S31N-AM2 at different pH. All data were obtained from VM+ membrane-bound M2 peptides, except for the pH 7.5 WT-BM2 data, which was measured in the POPC/POPG/cholesterol membrane [3]. The uncertainty in the percent populations is conservatively estimated to be ±5% based on differences between the 2D CC and NC results for GDR-BM2, and mainly reflects systematic uncertainty due to residual motion and different polarization transfer dynamics for different ^{13}C and ^{15}N sites.

**Peptides**	**pH**	**Experiment**	**Percent populations of histidine species**			
			**τ tautomer**	**π tautomer**	**[τ] : [π]**	**Cationic His**
GDR-BM2 (1-33)	7.5	2D CC (72 ms CORD)	73%	27%		0%
		2D NC	68%	32%		0%
		Average	70%	30%	2.3	0%
GDR-BM2 (1-33)	5.5	2D CC (72 ms CORD)	59%	22%		19%
		2D NC	55%	18%		26%
		Average	57%	20%	2.9	23%
WT-BM2 (1-33) [1]	7.5	2D CC (50 ms PDSD)	71%	30%	2.4	0%
	5.5	2D CC (100 ms PDSD)	62%	38%	1.7	0%
H27A-BM2	7.5	2D CC (70 ms PDSD)	41%	59%	0.7	0%
	5.5	2D CC (70 ms PDSD)	37%	41%	0.8	26±10%
S31N-AM2 (19-49) [1]	7.5	1D ^{13}C spectra	65%	25%	2.6	10%
	5.4	1D ^{13}C spectra	36%	12%	3.0	52%
WT-AM2 (22-46)[4]	8.5	2D CC	71%	29%	2.4	0%
	4.5	2D CC	0%	0%	n/a	100%
W41F-AM2 (22-46) [5]	7.5	2D CC (150 ms PDSD)	46%	54%	0.8	0%
	5.5	2D CC (150 ms PDSD)	0	0	n/a	100%

Table S4. Fractional intensities of the resolved ^{19}F peaks at acidic pH for three influenza M2 peptides obtained from spectral simulations. 5-^{19}F-Trp is labeled in all three peptides. The GDR-BM2 and WT-BM2 spectra were measured at pH 5.5 at 305 K whereas the AM2 spectrum was previously measured at pH 4.5 at 243 K [1].

States	^{19}F chemical shift range	GDR-BM2	WT-BM2	WT-AM2
State A	-124.0 to -123.8 ppm	57%	44%	28%
State B	-125.3 to -125.6 ppm	22%	14%	28%
State C	-126.8 to -127.2 ppm	21%	27%	44%
State D	122.7 ppm	0	15%	0

References

1. J. K. Williams, Y. Zhang, K. Schmidt-Rohr and M. Hong, *Biophys. J.*, 2013, **104**, 1698-1708.
2. B. Kwon, M. Roos, V. S. Mandala, A. A. Shcherbakov and M. Hong, *J. Mol. Biol.*, 2019, **431**, 2554-2566.
3. J. K. Williams, D. Tietze, M. Lee, J. Wang and M. Hong, *J. Am. Chem. Soc.*, 2016, **138**, 8143-8155.
4. F. Hu, W. Luo and M. Hong, *Science*, 2010, **330**, 505-508.
5. V. S. Mandala, S. Y. Liao, B. Kwon and M. Hong, *J. Mol. Biol.*, 2017, **429**, 2192-2210.
